# In situ ptychographic nanotomography captures activation, mobility, and deactivation of supported catalysts

**DOI:** 10.1038/s41467-026-73365-w

**Published:** 2026-05-28

**Authors:** Arik Beck, Mirko Holler, Tomas Aidukas, Andreas Menzel, Manuel Guizar-Sicairos, Jeroen A. van Bokhoven, Johannes Ihli

**Affiliations:** 1 ETH Zürich, Zürich, Switzerland; 2 Karlsruhe Institute of Technology KIT, Karlsruhe, Germany; 3 Paul Scherrer Institut, Villigen, Switzerland; 4 École Polytechnique Fédérale de Lausanne (EPFL), Lausanne, Switzerland; 5 ALBA Synchrotron, Barcelona, Spain

**Keywords:** Catalytic mechanisms, Heterogeneous catalysis, Imaging techniques

## Abstract

Nanoparticles supported on the surface of porous carrier materials are the dominant form of heterogeneous catalysts today. Yet, they suffer from a common deactivation mechanism: the loss of active surface area under industrial use conditions. Deactivation often stems from the sintering of nanoparticles, a mass-transport process whose mechanism and operating length-scale are a topic of controversy. Investigating this process is challenging, requiring not only a behavioral characterization of thousands of individual particles within the spatial confines of a hierarchically structured support but also a characterization of their ensemble behavior and local support interactions. Here, we introduce in situ ptychographic X-ray computed nanotomography as a tool to facilitate this characterization, allowing a local examination of catalysts in their use-geometry under operational-relevant conditions. Applied to methane oxidation over a palladium-on-silica supported catalyst, we reveal two concurrently operating deactivation drivers, short-range ripening and long-range particle migration, each with different temperature and atmosphere dependencies. The latter enables particles to traverse hundreds of nanometers through the support. These observations expand the current understanding of sintering behavior in supported catalysts and demonstrate PXCT’s capability to resolve restructuring processes within complex porous materials.

## Abstract (web summary)

Understanding and preventing the loss of active surface area in supported catalysts due to transport and sintering processes remains an industrial-scale challenge. Here, using in situ ptychographic nanotomography, the authors track the formation, mobility and aggregation of 100,000 s of catalyst nanoparticles in a supported catalyst under operating conditions.

## Introduction

Heterogeneous catalysts are central pillars of the chemical industry and essential to energy and chemical production as well as environmental remediation^1^. While certain levels of catalytic activity and selectivity are application requirements, industrial and economic viability is often determined by a catalyst’s longevity. Supported catalysts are a prime example of this, finding, for example, application in automobiles for the remediation of exhaust gases^2^. Here, catalytically active nanoparticles, often palladium, are finely dispersed on the surface of a porous support, and are used to transform unburned hydrocarbons and carbon monoxide to carbon dioxide^2^. During this transformation, and for years on end, the catalyst is exposed to high temperatures (> 400 °C), which results in a high catalytic activity, but gradually deactivates the catalyst^3^. This deactivation, as in many other catalysts, stems from the loss of active surface area^4^. In supported catalysts, this loss in surface area is typically caused either by Ostwald ripening, where smaller particles dissolve and redeposit onto larger ones, or by particle migration and coalescence (PMC) processes, where entire particles move across the support surface and then fuse^5,6^.

Characterization of these degradation processes and their prevention remains a major industrial-scale challenge. In-depth studies yet remain particularly difficult, requiring the observation of thousands of nanoparticles, <10 nm in diameter, within the confines of millimeter-sized, use-representative catalyst architectures and under application conditions^7^. Traditional, ensemble-averaging in situ techniques, such as X-ray diffraction and spectroscopy, remain invaluable for identifying bulk structural changes and average chemical states, but provide limited insight into local heterogeneities, spatial dependencies, and particle migration dynamics^8^. High-resolution imaging methods, in contrast, can provide these and allow for a more detailed understanding of catalyst behavior, including the mobility of individual nanoparticles^9–11^. For example, using in situ transmission electron microscopy (TEM), we showed that the nanoparticles in a supported catalyst can become highly mobile under reaction conditions, questioning a previously suggested pure and local Ostwald ripening driven deactivation^12^. Such TEM studies are essential to resolve atomic- to nanometer-scale mechanisms of sintering and metal–support interaction^5^. However, transmission electron microscopy is constrained to sub-micron sample volumes^5^ and commonly involves destructive sample preparation steps to obtain an electron-transparent specimen. This limits its use for studying realistic catalyst architectures and statistically representative particle behavior. This limitation becomes increasingly important as the focus shifts towards a holistic description of industrial catalysts, their function and deactivation, emphasizing the need for complementary, non-destructive approaches that probe structure and chemistry across representative sample volumes and over multiple length scales, thereby linking atomic-scale insights to mesoscale processes^4,13^. Ptychographic X-ray computed tomography (PXCT) is an emerging complementary method^14–17^, allowing us to measure such larger and intact sample volumes with sufficient spatial resolution to identify the nanoparticles within a supported catalyst^18^. Owing to missing instrumentation, PXCT has mainly been employed in an ex situ manner, i.e., measuring samples before and after catalytic reactions^16,19–23^. The removal of the catalyst from the reactor and its operational environment, however, alters the catalyst, making a use-representative characterization currently practically impossible.

Here, enabled by a custom and PXCT-compatible environmental control system^24^, we present an in situ ptychographic nanotomography study examining a supported palladium on silica (Pd/SiO_2_) catalyst across its complete lifecycle (activation–operation–deactivation). By repeatedly capturing the same volume from 25 °C to 830 °C under alternating methane-rich and oxidizing atmospheres, we quantitatively tracked the formation, growth, redistribution, and mobility of over 100,000 palladium nanoparticles.

The experiments revealed two distinct modes of palladium transport feeding catalyst deactivation. A short-range diffusion process consistent with Ostwald ripening, and a long-range particle redistribution mechanism, both leading to losses in surface area. Most strikingly, the experiments revealed that the majority of the palladium nanoparticles remain in motion during the reaction, showing that relocalization, fragmentation, and agglomeration are continuous processes. The experiments further allowed to quantify the average palladium transport velocity within the 3D pore structure, amounting to ~100 nm h^−1^ under the most severe operating conditions (20% O_2_/Ar at 750 °C). These mobility processes are strongly modulated by the gas environment, with purely oxidizing conditions promoting localized coarsening or ripening processes and methane oxidation conditions causing particle mobility across hundreds of nanometers. More broadly, this study highlights the ability of in situ PXCT to quantitatively resolve nanoscale transformations within large sample volumes and across environmental conditions. This bridges the resolution-statistics gap that has long stalled in situ characterization studies.

## Results

### Catalyst synthesis, performance, and deactivation

The palladium on silica (Pd/SiO_2_) catalyst was prepared by wet impregnation of a mesoporous support^25^ with a palladium nitrate solution^12,18,26^. The amorphous silica support possesses an average pore diameter of ~110 nm. The impregnation process results in the deposition of a thin film of palladium nitrate on the surfaces of the support, which, upon calcination, is converted into a dispersion of catalytically active PdO nanoparticles.

**Fig. 1:**
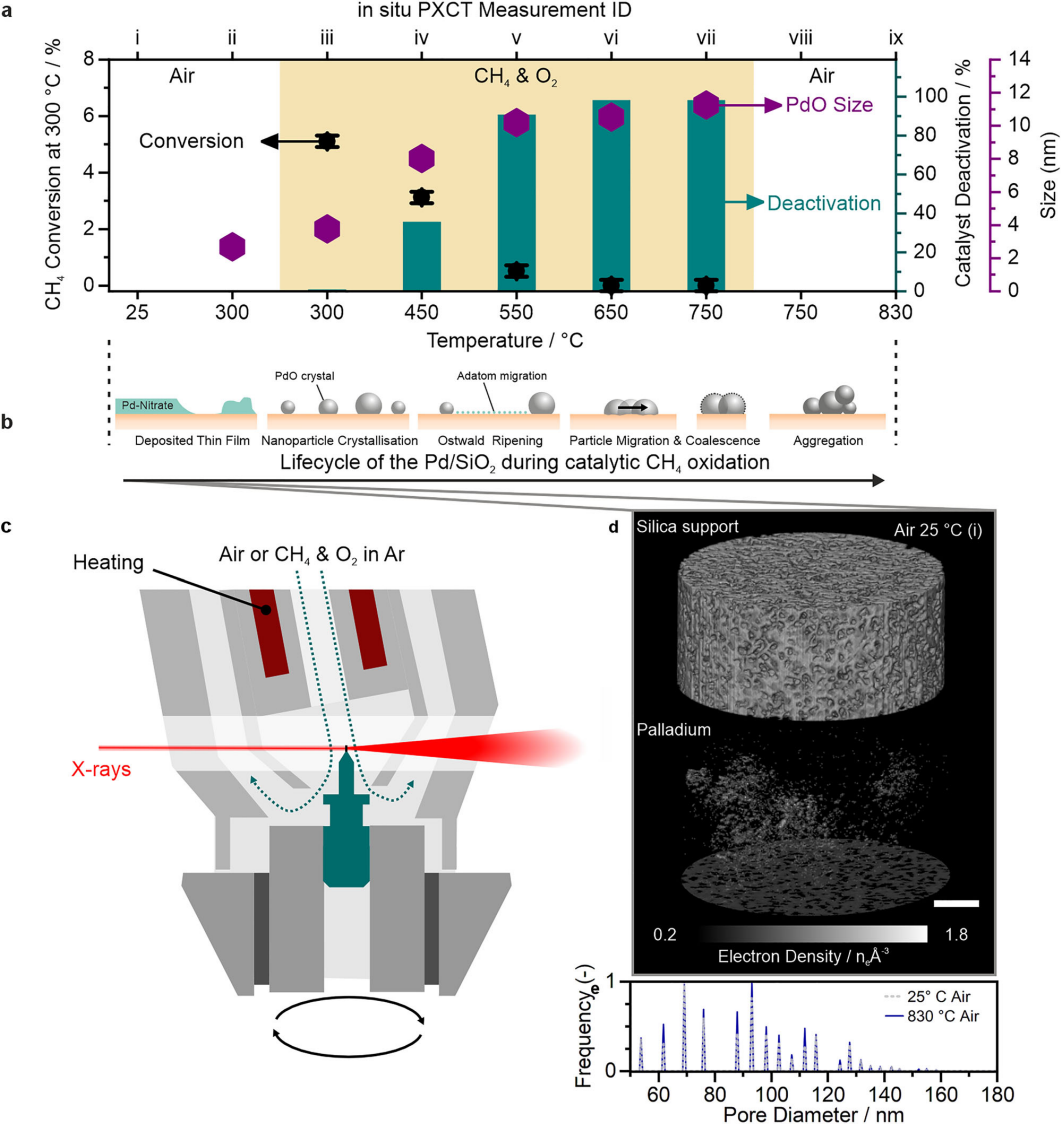
Linking methane oxidation performance with in situ PXCT of a Pd/SiO_2_ catalyst. **a** Temperature and atmosphere-resolved methane conversion, deactivation degree and averaged PdO size of a supported Pd/SiO_2_ catalyst. Plotted is the catalyst’s methane conversion level at 300 °C following consecutive 8-h operation windows at each of the indicated temperatures. The PdO size was determined ex situ from powder XRD using the Scherrer equation. Error bars for the conversion were determined for the standard deviation of conversion data recorded over 2 h. **b** Illustration of the structural evolution of the catalyst with temperature, starting from the deposition of a palladium-nitrate precursor thin film, its calcination and oxidation to form PdO nanoparticles, and their growth via several mass-transport mechanisms resulting in the catalyst deactivation. **c** Schematic of the in situ PXCT setup. Shown is a cross-section through the setup along the sample (turquoise)—X-ray path (red), highlighting the mechanical decoupling of the sample positioning and rotation instrumentation and the environment control system. The decoupling enables high-resolution in situ nanotomography over extended periods and across diverse environmental conditions. The catalyst is indirectly heated and insulated from the environment by a composition and temperature-adjustable gas stream. The green arrows indicate the gas in- and outflow directions. Matching the performance test, we examined a 7 µm wide catalyst sample as a function of temperature (25–830 °C) and atmosphere (Air or CH_4_ & O_2_). The acquisition sequence and the measured temperature-atmosphere combinations are provided in (**a**)—top *x*-axis. **d** In situ PXCT-derived and threshold-segmented electron density tomogram of the as-prepared catalyst sample. Shown exemplarily are the catalyst’s silica support structure (top) and the initial distribution of Pd-rich voxels (bottom). **e** Volume-weighted pore diameter distributions of the imaged catalyst pillar at 25 °C (i) and at 830 °C (ix).

To evaluate the catalyst’s methane oxidation activity as a function of temperature, the catalyst was then gradually heated to 300 °C (initiating precursor decomposition and PdO formation), 450, 550, 650 °C, and finally 750 °C under a constant flow of a methane-oxygen gas mixture (1 mol% CH_4_/4 mol% O_2_/94 mol% Ar) in a tubular reactor. Following a dwelling period of 8 h at each temperature, methane conversion was measured at 300 °C using mass spectrometry. The resulting activity profile (Fig. 1a) shows a gradual decline with increasing treatment temperature, indicating progressive catalyst deactivation.

Ex situ scanning transmission electron microscopy (STEM) and powder X-ray diffraction (PXRD) (Fig. 1a and S1–S3) show that this loss in activity correlates with an increase in the average size of the PdO particles, from ~5 nm in the as-prepared state to ~15 nm after treatment at 550 °C. Further, at this elevated temperature, larger sponge-like PdO agglomerates begin to appear. These observations suggest that the loss of catalytically active surface area is the primary cause of deactivation. However, ex situ measurements do not reveal the processes that lead to the agglomeration, especially the palladium transport process and its associated length scales and velocities.

### In situ ptychographic X-ray computed tomography

To capture those transport processes without destroying the pore architecture of the catalysts, we then examined a 7 µm-wide catalyst pillar (Fig. S4) via in situ ptychographic X-ray computed tomography^22,27^, capturing the evolution of the catalyst from nitrate-rich precursor decomposition to deactivation (Fig. 1b). Tomograms were sequentially acquired at 25 °C and 300 °C in air (calcination phase); at 300, 450, 550, 650, and 750 °C under a constant flow of the methane-oxygen gas mixture to mimic operating conditions; and again at 750 °C and 830 °C in air to investigate the effect of atmosphere on particle mobility and sintering (Fig. 1a). The tomograms have a voxel size of (15.5 nm)^3^ and a sample average spatial resolution of 35 nm (Fig. S5). A schematic of the acquisition setup is provided in Fig. 1c. Instrumentation and experimental details are provided in Holler et al.^24^ and in the “Methods” section. A key advantage of PXCT is that it provides quantitative electron density tomograms^15,16,28–31^, with each voxel returning an absolute density value. A priori knowledge of the electron densities of the catalyst’s components, ~0.00 n_e_Å^-3^ for air and argon, 0.55 n_e_Å^-3^ for amorphous silica and 2.21 n_e_Å^-3^ for PdO (Table S1), allows us then to segment and quantify material phases, respectively, track their evolution^32^. Fig. 1d shows a threshold-segmented volume rendering of the tomogram acquired at 25 °C, highlighting the silica support and the distribution of Pd-rich precursor deposits within it. The deposits are heterogeneously distributed and vary in density, reflecting the inherent limitations of wet impregnation processes, yielding here a volume-average Pd loading of ~1 wt.%.

**Fig. 2:**
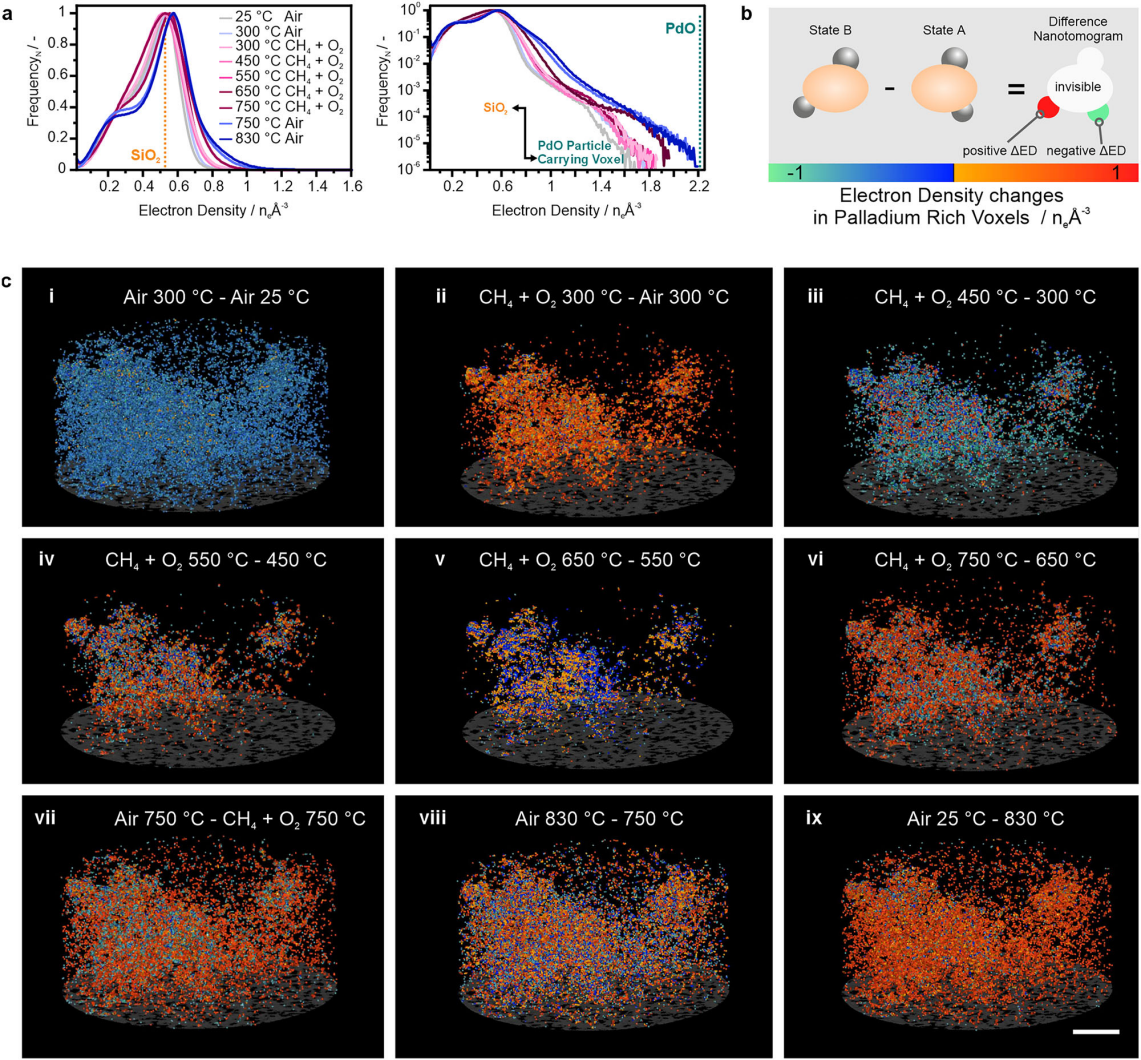
In situ ptychographic nanotomography of a Pd/SiO_2_ catalyst. **a** In situ tomogram extracted, and frequency-normalized electron density (n_e_Å^−3^) histograms. Shown is the gradual evolution of the catalyst structure and composition as a function of temperature and atmosphere. Provided is a linear (left) and log-scale (right) representation of the histograms. The nominal electron densities of the main catalyst’s components and the tomogram segmentation threshold (**-**) utilized in the classification of support and Pd-rich voxels are provided. **b** Concept of difference electron density tomograms. **c** Shown is a series of volume renderings depicting the location and evolution of Pd-rich or particle-carrying voxels in the examined sample volume. Specifically shown is the difference between adjacent environmental conditions. The color scale corresponds to the legend shown in (**b**). Increases in ED, corresponding to nanoparticle growth or movement to a specific location, are depicted using a color map ranging from orange to red. Conversely, decreases in ED, corresponding to disintegration or a particle migrating away from its original location, are depicted using a color map ranging from blue to green. The scalebar is 1000 nm.

A consolidated view of the catalyst’s evolution is provided in Fig. 2a, which shows the electron density histograms of the acquired tomograms. A first look reveals that the silica support is changing with temperature. Observable is a gradual increase in electron density from 0.53 n_e_Å^-3^ at 25 °C to 0.58 n_e_Å^-3^ at 750 °C. This densification, more pronounced in air, coincides with a net sample mass increase of ~7% (Fig. S6). Based on PXRD measurements, literature reports and the apparent high-defect density of the silica support, we currently attribute the observed densification to progressive elimination of volume defects, vacancies or silanol groups, whereas the concomitant mass gain is most likely due to oxygen uptake, e.g., the removal of oxygen vacancies and PdO formation (Fig. S7)^33,34^. Despite these density changes, we observed no spatial deformation of the support within our detection limits. Analysis of the segmented pore network shows that the pore size distribution remains essentially constant, with the average pore diameter decreasing only slightly from 113 at 25 °C to 107 at 750 °C (Fig. 1e & S8), i.e., well within the spatial resolution estimate of 35 nm and under a single voxel deviation of 15 nm.

A close examination of the histograms reveals that a dominant fraction of PdO particles remains below the voxel size, i.e., the voxel possesses an electron density <2.21 n_e_ Å⁻³, even at 830 °C. Although individual particle morphologies cannot be resolved at the current resolution, changes in electron density at the voxel-level provide a metric for quantifying PdO growth and mobility. An increase in voxel electron density reflects either particle growth or the aggregation of smaller PdO domains within that voxel, whereas a decrease indicates particle dissolution or relocation. Treating voxel electron density as a proxy for a volumetrically equivalent PdO particle size (Fig. S9 and Fig. 2a)^32^, we derive an average particle diameter of ~4.6 nm in the as-prepared catalyst, in agreement with the ~5 nm obtained by ex situ STEM (Fig. S2). This assignment is reasonable as palladium particles, whether metallic (Pd) or oxidized (PdO), are the densest and mobile component in the system, meaning that observed density changes can be attributed unambiguously to palladium dynamics. Now, focusing on these Pd-rich voxels, defined here as those exceeding a density of 0.80 neÅ⁻³, or containing ≥15 vol.% PdO (Fig. S9), we observe a steady increase in their number and average density (or effective particle size) with increasing temperature. Based on the changes of the histograms, the formation of PdO particles is initially driven by precursor consumption, while at higher temperatures their continued growth involves coalescence and long-range migration, with increasingly isolated PdO domains growing at the expense of smaller ones. Surprisingly, upon switching from the methane oxidation atmosphere to air at 750 °C we observe a marked collapse of the left-side tail of the silica peak in the histogram and a concurrent rise in Pd-rich voxels. We believe this change is caused by oxidation-driven reorganization of previously porous, fragmented, or partially reduced palladium particles into more densely packed and well-defined PdO particles.

Building on the histogram analysis of the Pd-rich voxels, we visualized their spatial distribution as a function of temperature and atmosphere. Figure 2b and Movies S1 & S2 present volume renderings of the electron density differences between adjacent environmental conditions. This subtraction removes the constant contributions of the support and pore space, thereby highlighting locations where palladium accumulates or relocates to within the sample volume.

**Fig. 3:**
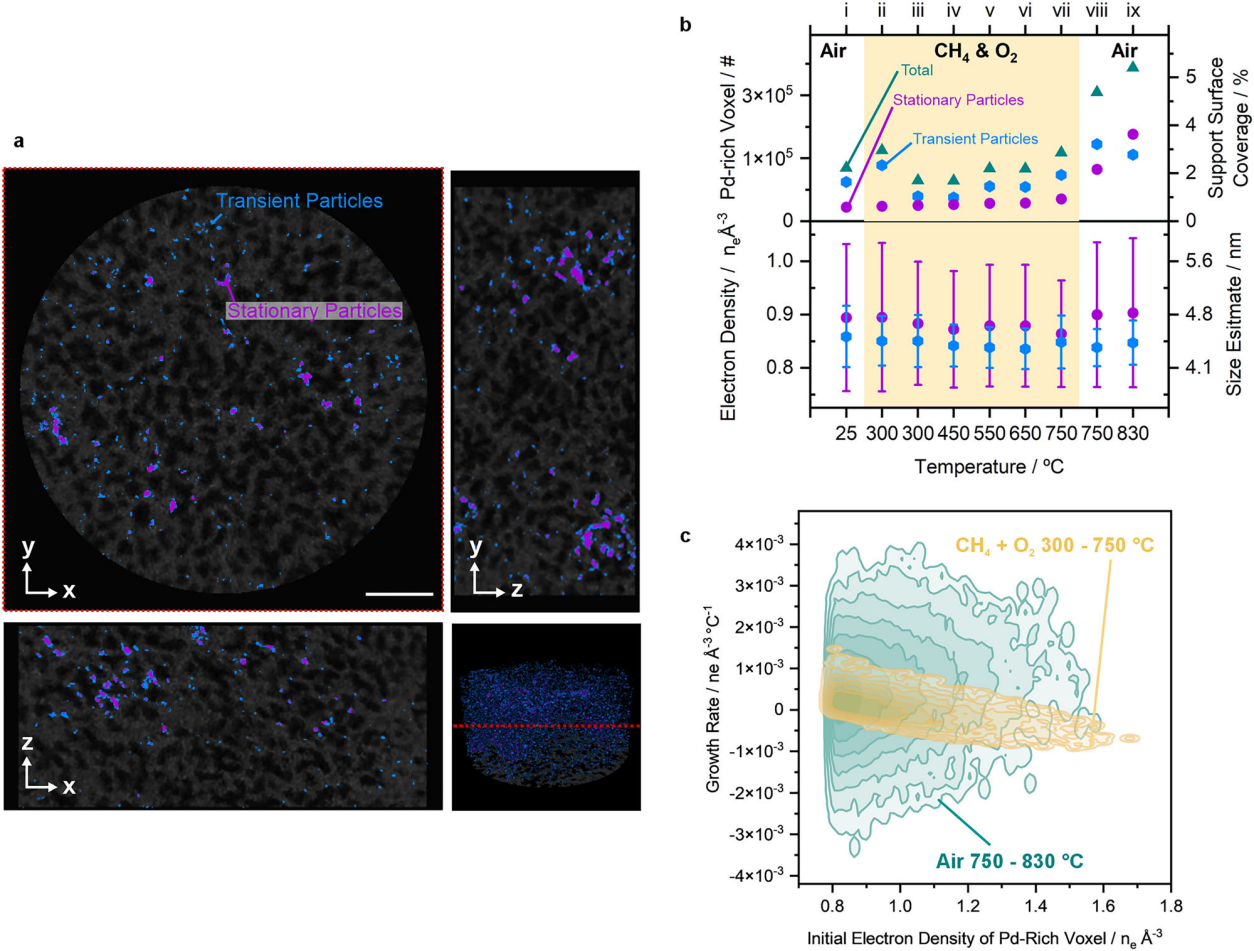
Mobility of palladium particles in the supported catalyst. **a** Provided are virtual cuts through the 4D tomogram showing the location of voxels permanently (stationary particles) and temporarily (transient particles) occupied by palladium particles. Voxels that consistently display an electron density greater than 0.8 n_e_Å^−3^ from the first temperature at which this threshold was reached are classified as permanently occupied by palladium particle(s), the remainder as temporarily occupied. Locations are shown on top of the silica support (gray). The scalebar is 1000 nm. **b** Analysis of the permanently and temporarily occupied voxel. Plotted is the number of stationary and mobile palladium particles as a function of temperature and atmosphere. Further provided the evolution of the population-average particle diameter, both for the stationary and mobile palladium particles. The error bars represent the measured electron density standard deviation. **c** Kernel density plots showing the voxel-level growth rate (n_e_Å^−3^ °C ^-1^) distribution of a stationary Pd-rich voxel ( > 0.8 n_e_Å^−3^) as a function of initial particle size or electron density. The relationship is plotted for methane oxidation and the purely oxidizing operation windows. Included are particles initially present in the sample and those that have formed at elevated temperatures. Growth rates represent a linear fit across the considered temperature range.

Prominently visible in Fig. 2c (i)—showing the difference between the as-prepared catalyst and the catalyst after activation at 300 °C in air—is the decomposition of the deposited precursor and the formation of PdO particles; a first growth process. Continued treatment at 300 °C under methane oxidation conditions (ii) leads to a notable increase in the number of Pd-rich voxels across the support, visually resembling a typical supported catalyst. Importantly, no significant electron density loss is detected elsewhere, indicating that particle formation arises from redox-driven densification of highly dispersed Pd-rich material into stable PdO nanoparticles rather than the coarsening of visible crystallites. Given the relatively low temperature and the presence of both oxidizing and reducing gases, partial redox cycling (Pd^2+^/Pd^0^) may facilitate redistribution and nucleation at this stage^35^. These processes precede the onset of classical and here visually detected Ostwald ripening or PMC^5,36^, which becomes increasingly evident at higher temperatures (iii), where particle growth is accompanied by the shrinkage and or disappearance of smaller particles in localized regions. (iv–vi) The stepwise examination up to a temperature of 750 °C under methane oxidation conditions showed a gradual but limited increase in both particle size and number consistent with continued coarsening. However, this stage is also marked by abrupt shifts in the spatial distribution of Pd-rich voxels, suggesting the presence of an additional, longer-range palladium redistribution mechanism. The emergence of new Pd-rich domains, often hundreds of nanometers away from regions of simultaneous depletion, points to palladium relocation via the long-range migration of particles, aggregates, or fragments. In parallel, we further observe the emergence of large, spatially fixed Pd-rich agglomerates, often porous and spanning multiple voxels, which appear to condense from this redistributed material. (vii-viii) Continued heating to 830 °C and, importantly, a change to a purely oxidizing atmosphere appear to suppress this long-range redistribution. Instead, we observe a distinct increase in newly resolvable Pd-rich voxels (Fig. 3c). This behavior may reflect a transition in dominant growth or transport mechanism. Under methane-rich conditions, limited surface mobility is observed while selected palladium particles remained mobile, enabling long-range redistribution. In contrast, under fully oxidizing conditions, surface and gas-phase diffusion of PdO molecules and local restructuring are enhanced, resulting in an increase in Ostwald ripening-dominated growth^37^.

### Quantification of Pd transport processes

To investigate possible spatial and mechanistic dependencies of these transport processes, we identified the location of mobile and stationary Pd-rich voxels. Figure 3a shows the location of permanently and temporarily occupied regions—defined here as voxels that showed a Pd signal consistently or intermittently across the in situ measurement. The permanently occupied voxels (stationary particles) are found preferentially near pore throats. Temporarily occupied voxels (transient particles) appear predominantly at the outskirts of stationary particle agglomerates or near open pore channels (Movies S3 & S4). Analysis further revealed a temperature-dependent increase in both voxel populations (Fig. 3b), reflective of the thermally activated PdO particle formation and mobility. Mobility is also influenced by both particle size and atmosphere (Fig. 3c). While stationary particles are on average only ~2 nm larger than mobile ones, no particle with an inferred or volumetrically equivalent diameter >9 nm was observed to be mobile (Fig. S10). The observed size-dependent mobility is consistent with models of particle transport, in which surface diffusion or gas-phase migration becomes increasingly restricted with particle size due to increasing particle-support interactions^8^.

Further analysis of the stationary voxels revealed a non-monotonic growth behavior, i.e., the same voxel might record gains in electron density or Pd content across consecutive temperatures just to display a loss in the next. A behavior captured in the voxel-level growth rate distributions shown in Fig. 3c, plotted here as a function of initial electron density for both the methane oxidation (300–750 °C) and oxidizing (750–830 °C) operation windows. Under methane oxidation conditions, we observed a relatively narrow distribution, with positive growth rates in smaller particles and an initially unexpected net loss in larger ones. A likely result of fragments or particle hopping events seen in Fig. 2b(iv–vi). Upon switching to a purely oxidizing atmosphere, the growth rate distribution becomes broader and overall positive, with enhanced growth for smaller particles.

**Fig. 4:**
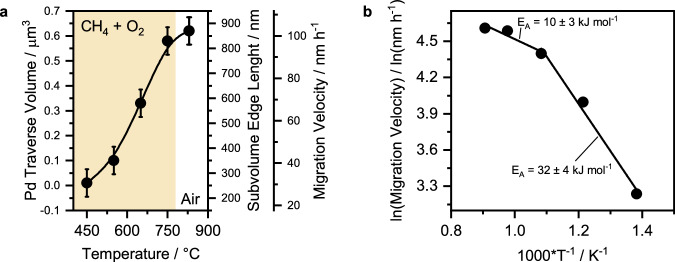
Transport length and migration velocity. **a** Palladium transport volume, correlated subvolume edge length, and migration velocity as a function of temperature and atmosphere. Error bars represent the standard deviation. **b** Derived Arrhenius-type plots for the migration velocity. Details on the calculation of the traverse volume by subvolume sampling can be found in Supplementary Fig. S12. In short, a central volume was iteratively sub-sampled into progressively smaller sub-volumes. For each subvolume, the integrated electron density change (ΔED) across a defined temperature window was then calculated. The maximum Pd displacement length is inferred from the smallest subvolume size at which a ≥ 5% deviation in normalized ΔED appears, indicating non-local Pd redistribution.

Finally, to determine the palladium transport length and migration velocity through the support, we applied a fractional volume subsampling approach (Fig. 4a and Fig. S12). By calculating the net electron density change within progressively smaller sub-volumes, we identified the volume size (Pd traverse volume) below which mass redistribution becomes detectable. For volumes larger than the palladium transport length, density changes average out; below this threshold, local gains or losses become visible. The edge length of this traverse volume serves as a proxy for the maximum transport length (subvolume edge length). This analysis reveals a temperature-dependent increase in transport length under methane-rich conditions: from ~200 nm at 450 °C to ~830 nm at 750 °C. Heating the air to 830 °C further enhances the distance to 850 nm. Using these transport lengths together with the tomogram acquisition time, we can approximate the average palladium migration velocities, which increase from ~30 nm h^-1^ to ~105 nm h^-1^ (Fig. 4b). These values provide a kinetic measure of palladium redistribution and are critical for the prediction of catalyst stability and industrial catalyst body design. The velocities reveal a strong exponential temperature dependence. Accordingly, Fig. 4c presents an Arrhenius-type plot with two distinct regimes. In air, at higher temperatures, the apparent transport activation barrier is low (~ 10 kJ mol^-1^) and matches previous studies that have shown a size-independent gas-phase transport of palladium leading to strong and rapid sintering in Pd/SiO_2_ catalysts^37^. Under methane oxidation conditions, a substantially higher barrier of 32 kJ mol^-1^ is found, reflecting the distinctly different migration/transport processes.

## Discussion

In situ ptychographic nanotomography enabled us to monitor the evolution of a supported Pd/SiO_2_ catalyst for methane oxidation across a full activation–operation–deactivation cycle, from room temperature up to 830 °C under oxidizing and methane oxidation atmospheres. The tomograms, encompassing hundreds of thousands of individual catalyst particles, reveal a complex and environment-dependent picture of catalyst activation, particle mobility and deactivation.

During activation (25–300 °C), PXCT reveals the decomposition of the palladium nitrate precursor and the formation of finely dispersed PdO nanoparticles, which are non-uniform in size and heterogeneously distributed across the silica support. Under operational methane oxidation conditions (300–750 °C), two particle growth and mobility regimes are detectable. At lower temperatures, the particle growth is dominated by classical Ostwald ripening and PMC processes. Nanoparticles increase gradually in size, initially through the consumption of residual precursor material or particles with a size below the detection limit, and subsequently via the coalescence or aggregation of smaller particles. At higher temperatures, an unexpected long-range palladium redistribution process becomes dominant. This is evident by the appearance and disappearance of PdO particles at spatially separated sites on the support, with inferred transport lengths reaching up to 800 nm at 750 °C. This long-range migration of what looks to be entire particles exceeds the typical surface diffusion lengths, and importantly, migration does not always lead to immediate sintering. Instead, some Pd species appear to remain mobile without coalescing, while others condense into porous aggregates. Upon switching the sample atmosphere to air at 750 °C, regardless of further heating to 830 °C, the particle dynamics change markedly. The long-range mobility is suddenly strongly suppressed, and particle growth becomes again more localized. Additionally, larger PdO particles emerge via renewed ripening, consistent with an increased surface diffusion length under oxidizing conditions. This enhanced mobility essentially allows previously sub-resolution, or porous palladium species to migrate across the support and condense into stable, well-defined and now detectable nanoparticles. These findings indicate two distinct transport and deactivation pathways for Pd on SiO_2_ supports under oxygen-rich conditions: (1) a short-range, surface-mediated coarsening process, e.g., a combination of Ostwald ripening and PCM, that governs initial nanoparticle formation and growth, and (2) a long-range particle redistribution mechanism that operates beyond the scale of conventional surface diffusion.

The short-range process is both spatially confined and chemically modulated. Even at elevated temperatures, particle diameters rarely exceed ~15 nm, with numerous sub-6 nm PdO particles persisting adjacent to larger aggregates (Fig. 4 and S1)—sometimes separated by only one to two voxels—rather than merging. Assuming full precursor support surface coverage, the dominant absence of particles larger than ~15 nm or the diameter of a voxel allows us to estimate the effective surface mobility to be ~30–60 nm. This is consistent with literature reports of limited (< 100 nm) surface mobility on oxide supports^38^. We attribute this confined transport length to a chemical stabilization or strong-surface interaction under methane-rich conditions, rather than topological barriers of the support. Upon switching to a purely oxidizing atmosphere, the number of resolvable PdO voxels increases sharply, consistent with enhanced mobility driven by gas-phase–mediated Ostwald ripening at high temperatures^37,39,40^. This limited transport length and the associated growth of particles are yet sufficient to deactivate the catalyst, as the resulting decrease in surface-to-volume ratio drastically reduces the catalytically active surface area.

The long-range redistribution of PdO particles is more active under methane-rich conditions. Between 300 °C and 750 °C, Pd-rich voxels appear and disappear across distances of hundreds of nanometers between tomogram acquisitions, far exceeding the expected range of surface diffusion. We speculate that entire nanoparticles or small clusters detach from the support, migrate through the connected pore space and re-adsorb in new locations and eventually cluster. Based on the apparent suppression of this transport under fully oxidizing conditions, this particulate migration may be driven by redox-induced changes in metal–support adhesion, temporarily “unlocking” particles so they can traverse the pore network^41^. We currently speculate that heterogeneity of the amorphous oxide support surface^35,42,43^, as well as possible gas-phase inhomogeneities, particularly gradients in local product concentrations (e.g., H_2_O)^44^ under methane oxidation conditions, may influence which particles detach from the support and become mobile or remain stable. Thus, modifying the surface by strongly binding an anchor has been shown to stabilize metal particles in place for methane oxidation^41^. Similar evidence for cluster mobility has been reported for Pt/TiO_2_ under reactive atmospheres, suggesting that long-range particle migration may be a general phenomenon in harsh catalytic environments^12^. Furthermore, we obtained a first estimate of the velocity of long-range PdO migration, which is strongly temperature and atmosphere-dependent and ranges from 20–100 nm h^-1^. To put this into perspective, real-life catalyst bodies are typically millimeter-sized: for instance, at a migration rate of 100 nm h^-1^ a PdO particle would require ~1.1 years to traverse 1 mm through the support. A time scale well within the common lifetime of several years for many heterogeneous catalysts^45,46^.

In combination, these observations not only query the optimal strategies to prevent catalyst deactivation but also challenge the notion that nanoparticles are inherently immobilized or only weakly mobile on support surfaces. Accordingly, strategies that rely solely on optimizing interparticle distances or initial dispersion patterns^47,48^ may prove insufficient to prevent deactivation under such conditions. Instead, more comprehensive approaches are needed: anchoring particles through tailored metal–support interactions ^41,49,50^ and designing support architectures that physically restrict mobility, e.g., via tortuosity engineering or controlled confinement, may offer more robust stabilization under harsh conditions.

While quantitative in situ nanotomography facilitates a more representative and holistic characterization of heterogeneous catalysts, the approach is currently bounded by spatial and temporal resolution as well as chemical specificity. These limitations currently prevent us from directly probing local metal–support interactions^26,51,52^, i.e., probing the morphology and chemistry of individual particles and the surrounding support, or the kinetics of migration events at the single-particle level. For example, while ex situ PXRD and PXCT indicate only the presence of PdO throughout, PdO is known to autoreduce above 600 °C to metallic palladium in oxygen-rich atmospheres^53^. Although such transient metallic phases are likely minor in terms of overall composition, their possible presence and relevance highlight the need for greater chemical specificity and sensitivity. Ongoing advances in instrumentation^24,54–57^, dynamic tomography^22,58,59^, and chemical imaging^21,22,29,60,61^ will alleviate these limitations in the near future. Follow-up experiments at a 4th generation synchrotron^56^, will enable combining dynamic PXCT^62^ with X-ray absorption spectroscopy^22^, allowing real-time, chemistry-specific tracking of particle evolution and metal–support interactions at previously inaccessible scales.

This study presents the first demonstration of in situ ptychographic nanotomography of a heterogeneous catalyst. By monitoring the evolution of over 100,000 individual catalyst particles in a geometry representative of their actual usage and under conditions that approach their real-world application, we revealed that Pd/SiO_2_ catalyst deactivation under methane oxidation and calcination conditions is governed not only by localized coarsening via ripening and coalescence but also by long-range redistribution of entire nanoparticles across hundreds of nanometers. This atmosphere-dependent mobility revises the picture of nanoparticle stability and highlights the need for strategies that anchor particles and engineer supports to restrict migration. The ability to bridge or even narrow the information gap between in situ electron microscopy^3,12^ and the statistical relevance of averaging in situ methods^63^ is further expected to provide new, length-scale, overarching insights into heterogeneous catalysis. Importantly, the presented approach is not limited to catalysis: it is applicable to any reactive porous material, including battery electrodes, fuel cells, and degradation-prone nanocomposites, laying the groundwork for more predictive materials design across disciplines.

## Methods

### Material synthesis

Nanoporous glass beads were obtained from the *Bundesanstalt für Materialforschung und—Prüfung* (BAM) and used as the catalyst support matrix. The beads possess an average pore diameter of 139 nm and a specific surface area of 26.6 m^2^g^-1^ (refs. ^25,30^). Analytical grade palladium nitrate dihydrate, Pd(NO_3_)_2_ 2H_2_O, was purchased from Sigma Aldrich. The synthetic gas mixture (1 mol.% CH_4_/4 mol.% O_2_/94 mol.% Ar) was purchased from Carbagas.

#### Palladium deposition/supported catalyst preparation

Pd/SiO_2_ catalysts were prepared via an incipient wetness impregnation process. Specifically, 1 g of the glass beads was first immersed in a Pd(NO_3_)_2_ solution (12.7 mg mL^-1^) prepared using MiliQ water. The resulting slurry was next transferred into a vacuum desiccator to fully infiltrate the pores with the Pd solution. To then anchor the Pd species on the support surface and evaporate water, we removed the beads from the solution and placed them into a preheated oven for 10 min at 200 ˚°C. This process was repeated 6 times, yielding a Pd/SiO_2_ catalyst with a theoretical Pd loading of 2.9 wt.% based on a monolayer coverage in each cycle. A comparison of the integrated electron density between tomograms of the bare and loaded support suggests an average Pd loading of ~1 wt.%, assuming the density increase results solely from the added precursor. The lower-than-expected value suggests that precursor deposition was incomplete and uneven across the support. This is supported by Fig. 1d, which shows extended regions where no Pd signal is detected.

### General material characterization

#### Catalytic activity tests

Methane oxidation tests were conducted using a combined microreactor and mass spectrometry system (Hiden Analytical), consisting of a heated quartz reactor tube (3 mm inner diameter) coupled to a mass spectrometer. For each experiment, 10 mg of the supported catalyst (crushed beads, 125–250 µm in diameter) was loaded between two quartz wool plugs inside the reactor. The reactor was flushed with helium (50 mL min⁻¹), and the catalyst was then heated to 300 °C at a rate of 5 °C min⁻¹ under helium flow. Upon reaching 300 °C, the catalyst was exposed to a reaction gas mixture containing 1 mol% CH₄, 4 mol% O₂, and 94 mol% He at a total flow rate of 50 mL min⁻¹ for 8 h, corresponding to the PXCT tomogram acquisition time. Under these conditions, carbon dioxide and water were the only detectable reaction products. Activity or Methane conversion degree was calculated as stated in Eq. (1):

1 $${Conversion}=\,1-\,\frac{{\left[{{CH}}_{4}\right]}_{{out}}}{{\left[{{CH}}_{4}\right]}_{{in}}}$$

In follow-up experiments, the same protocol was applied with additional thermal ageing steps. After the initial 300 °C exposure, the catalyst was heated to 450 °C and held under reaction conditions for 8 h. It was then cooled to 300 °C, and the catalytic activity was re-evaluated. This procedure was repeated for 550, 650, and 750 °C treatments. Catalyst deactivation was calculated from the loss in conversion at 300 °C after each thermal step, as described in Eq. (2):

2 $${Deactivation}=\,1-\,\frac{{Conversion}\,{at}\,300\,^\circ {C}_{{after}\,T\,{step}}}{{Conversion}\,{at}\,300\,^\circ {C}_{{initial}}}$$

Following each test cycle, a fraction of the spent catalyst was retrieved and analyzed by STEM and XRD to track nanoparticle sintering, phase evolution, and structural degradation associated with deactivation.

#### Powder X-ray diffraction (PXRD)

Powder X-ray diffraction (PXRD) data were acquired using a Cu-K alpha radiation source with a scan step size of 0.02 2theta (Fig. S3). Instrument broadening was accounted for.

#### Electron microscopy

Scanning electron micrographs (SEM) were acquired using a Zeiss NVision-40. The scanning transmission electron micrographs (STEM) were acquired with an aberration-corrected Hitachi HD-2700CS microscope operated at 200 kV. High-angle annular dark-field (HAADF) images were acquired to facilitate compositional and morphological interpretation.

### Operando ptychographic tomography

#### Tomography sample preparation

To prepare the PXCT-examined sample pillar, we mounted a single, millimeter-sized, empty glass bead on top of an aluminum tomography pin using a carbon adheasive^64^. The pin was then placed in a furnace preheated to 200 °C and held for 90 min to fully cure the adhesive. After curing, the bead was shaped into a cylindrical pillar ( ~ 30 µm diameter, ~50 µm height) using a micro-lathe^27^. The upper portion of this pillar was subsequently thinned to ~7 µm in diameter via focused ion beam (FIB) milling (Supplementary Fig. S4). The resulting glass pillar was then subjected to the catalyst preparation procedure outlined above.

Ptychographic X-ray computed tomography (PXCT) is the combination of ptychography and X-ray computed tomography^14–16^. Ptychography is a lensless coherent imaging technique where the sample is scanned across a coherent X-ray beam, and a diffraction image is acquired at each scanning position. By ensuring that the neighboring beam illuminations overlap with each other, the resulting redundancy encoded within the diffraction images enables the reconstruction of both the sample and the X-ray beam through iterative optimization algorithms^16^. By performing ptychography at multiple sample rotation angles, the resulting ptychographic projections enable tomographic reconstructions of both phase and amplitude contrast with nanometer resolution, ideal for local component identification in functional materials^20,22,31,32^. The phase tomogram, when acquired away from sample-relevant X-ray absorption edges, can be converted to an absolute electron density tomogram^31^.

PXCT^14,15^ experiments were carried out at 6.2 keV at the cSAXS beamline of the Swiss Light Source, Paul Scherrer Institut, Switzerland. The photon energy was selected using a double-crystal Si(111) monochromator. The horizontal aperture slits, located 22 m upstream of the sample, were set to 20 μm in width, to create a horizontal virtual source point that coherently illuminated a Fresnel zone-plate, 200 μm in diameter, with an outermost zone width of 60 nm and a focal length of 60 mm. The Fresnel zone-plate was designed with locally displaced zones to improve imaging quality^64^. The sample was placed 1 mm downstream of the focus, resulting in an illumination probe size of 4 µm. Coherent diffraction patterns were acquired using an in-vacuum Eiger 1.5 M area detector, with a 75 µm pixel size, placed 5.225 m downstream of the sample inside an evacuated flight tube. For sample positioning and environment regulation, we used the flexible tOMography Nano Imaging endstation (flOMNI)^30,65^ with modifications for environmental control^24^. For achieving fast scanning speeds, a Fresnel zone-plate scanner utilizing a combined motion of sample and illuminating FZP was used^66^.

The field of view of each projection, or ptychographic scan, varied from 10 × 5 μm^2^ to 15 × 12.5 μm^2^ (width x  height). The sample was scanned using a Fermat-spiral scanning pattern with an average step size of 0.7 µm, resulting in around 350 scanning points per projection. The exposure time per scanning point was 0.05 s. Total acquisition time per projection, including positioning overhead, was around 30 s. For each ptychographic scan or image reconstruction, a detector region of 900 × 900 pixels was used, resulting in a reconstructed image pixel size of 15.48 nm. Reconstructions were performed using the PtychoShelves package^67^ with 500 iterations of the difference map algorithm^14^ followed by 600 iterations of maximum likelihood refinement^68^. All the tomograms mentioned in the main text were reconstructed using ~550 ptychographic projections, sufficient for a tomogram spatial resolution of 20 nm for a 7 µm (non-porous) sample according to the Crowther criterion. Tomography projection acquisition followed a nested approach: we acquired 30 equally spaced projections over 180° at a time, then repeated the acquisition with an angular offset based on the golden ratio. The approach was chosen to accommodate possible sample stabilization delays. Following ptychographic image reconstruction and alignment of the projections, tomograms for each environmental condition were reconstructed separately by first aligning the projections^69^, followed by tomogram reconstruction using a modified filtered back-projection algorithm (FBP)^70^.

To investigate nanoparticle formation, mobility and catalyst deactivation, we examined the prepared sample pillar across 9 environmental conditions. In sequence, we examined the pillar (1–2) at 25 °C and 300 °C under a constant flow of air, (3–7) at 300, 450, 550, 650, 750 °C under a constant flow of a methane gas mixture (1 mol.% CH_4_/4 mol.% O_2_/94 mol.% Ar), and (8–9) at 750 °C and 830 °C under a constant flow of air. See Supplementary Fig. 1 for a graphical representation of the tested conditions and associated catalyst performance. Using the environmental control system^24^, integrated mass flow controllers, we ensured that the gas flow across measurements was kept constant. The flow rate was set to 1 L min^-1^. While tomogram acquisition commenced after a 30-min wait period after each change in environmental condition, we excluded the 6 initially acquired sub-sets of projections from the tomogram reconstruction of a given environmental condition. This exclusion introduced a sample stabilization or thermodynamic equilibration time of ~2.0 h per condition and tomogram acquisition.

#### Tomogram dose estimation

The imparted X-ray dose during a single tomogram acquisition was estimated to be on the order of ~10^8 ^Gy. The estimated dose is based on the average area flux density of each ptychographic scan and the mass density of the specimen^71^. For this calculation, the sample was assumed to consist entirely of silica.

#### Tomogram spatial resolution evaluation

Tomogram analysis was performed solely on the phase component of the acquired PXCT datasets, due to its superior spatial resolution and signal-to-noise ratio at the selected X-ray energy^61,72^. Spatial resolution estimates for each of the reconstructed tomograms were obtained using Fourier shell correlation (FSC)^73^. The acquired projections per conditions were alternatively separated into two sets and individually reconstructed. To estimate the *tomogram average* spatial resolution, we then calculated the correlation function between these two tomograms in the Fourier domain. The achieved resolution is estimated based on the first intersection of the correlation function with the half-bit threshold criteria (Fig. S5). The tomogram spatial resolution, averaged across all environmental conditions according to FSC, is 35 nm.

#### Voxel-level electron density uncertainty

To estimate the error of the retrieved electron density, we calculated the standard deviation (*σ*) of a region of air/argon surrounding the imaged pillar. Assuming the region to possess a uniform known density. The average electron density uncertainty was calculated to be ~0.008 n_e_Å^-^³.

### Tomogram analysis

Tomogram analysis and 3D rendering were performed using in-house developed Matlab routines or using Avizo. Prior to analysis, the tomograms were registered via a subpixel image registration approach using a mutual information metric and resampled onto a common grid. Tomography analysis was limited to a common sample volume present in all acquired tomograms^24^. Further, to exclude possible FIB sample preparation artifacts from the tomogram analysis, we excluded the outermost micrometer of the imaged sample cylinder from analysis.

#### Pore analysis

Isolated pores and pore networks were identified through threshold-based segmentation of the electron density tomograms. An upper electron density threshold of 0.28e⁻Å⁻³ was applied. The threshold, selected in view of the electron density of the silica support (0.56 n_e_Å^-^³ Table S1), classifies all voxels that are at a minimum filled with 50 vol.% with air or argon as pores. Silica is the lightest material in the sample. The isolated volumes were then subjected to a morphological closing operation to refine the segmentation, followed by 3D thickness map calculations to retrieve a pore size distribution for each tomogram/environmental condition. Shown in Supplementary Fig. 8 are a subset of the obtained, volume-weighted, pore diameter distributions. Pores with diameters below 50 nm were excluded from the thickness map analysis.

#### Effect of the wet impregnation process on the silica support structure

To evaluate whether the Pd impregnation process altered the silica support structure, and to establish a baseline for histogram interpretation and Pd feature identification, a tomogram of the pure silica support material was acquired prior to catalyst loading. Figure S13 presents a comparison of electron density histograms extracted from tomograms of the silica support before and after wet impregnation.

#### Extraction and analysis of environment-induced sample changes

Extraction and Analysis of Environment-Induced Sample Changes: Following the spatial registration and resampling of the tomograms onto a common grid, we segmented the sample into two principal components: the silica support and voxels at one point occupied by Pd across the measurement series. To isolate the silica support from the pore space, we applied a lower threshold of 0.28 n_e_Å^-^³ and an upper threshold of 0.8 n_e_Å⁻³. Values were informed by the electron density histogram of the bare support (Fig. S13). Voxels with densities exceeding 0.8  n_e_Å⁻³ were classified as Pd-containing, corresponding to a minimum voxel occupancy of 15 vol% PdO (Fig. S9). This separation allows us then to monitor evolution, both support and the growth and mobility of already existing or newly forming nanoparticles.

(1) Silica support changes and metal–support interactions: Having isolated the support in all tomograms, we first confirmed that the silica support did not undergo spatial deformation on the observable level (Table S1 and Fig. S8—Pore Size Distribution) over the course of the measurement series. This justifies the use of a constant support mask for the succeeding Pd nanoparticle analysis steps. While no spatial deformation of the support was detected, minor electron density changes of radial and environmental dependency were detectable (Figs. S6 and 7^22^). We currently believe these changes stem from the removal of oxygen vacancies from the silica support.

(2) Pd nanoparticle formation, growth, and migration: To track the evolution of Pd particles across the measurement series, we first generated a “Pd location mask” by summing all segmented tomograms. This produced a binary map identifying all voxels that contained Pd at any point during the experiment. The resulting mask was then applied to all tomograms to enable voxel-wise analysis across conditions. To provide a first qualitative impression of particle growth and mobility, we subtracted tomograms acquired under adjacent environmental conditions (e.g., subtracting the 450 °C tomogram from the 550 °C tomogram). The resulting differential maps, visualized using a divergent colormap scaled across the full dataset, highlight regions of Pd accumulation and depletion (Fig. 2 and Movie S1). For quantitative analysis (Fig. 3 and Movie S3), we evaluated the electron density of each Pd-containing voxel individually across the series. This voxel-level analysis allowed us to determine growth and dissolution rates, as well as the fraction of stationary versus transient Pd-occupied voxels. Being a voxel-level analysis, and as a dominant fraction of the initially present and newly forming Pd particles are smaller than the spatial resolution, growth rates here refer to net Pd density fluctuations within individual voxels.

(3) Transport length: To estimate the volume or distance over which Pd can migrate within the support under the tested conditions, we employed a volume-subsampling approach. A central volume of approximately 40 µm³ was used for this analysis. The previously defined Pd-location mask was applied to this volume to exclude contributions from the silica support. For each environmental condition in the measurement series, we calculated the integrated electron density within this volume. By incrementally subsampling this region into smaller nested volumes and performing the same integration, we identified changes in the rate of electron density gain or loss as a function of volume size. The key assumption is that, for a fixed condition, the rate of Pd accumulation or depletion should remain constant across nested volumes—unless Pd species begin migrating into or out of that volume. A deviation in growth rate exceeding ±5% between adjacent sub-volumes was used as a cut-off to define the maximum effective transport distance under each condition.

## Supplementary information

Supplementary Information

Description of Additional Supplementary Files

Supplementary Movie 1

Supplementary Movie 2

Supplementary Movie 3

Supplementary Movie 4

Transparent Peer Review file

## Data Availability

Data needed to evaluate the presented conclusions are provided in the manuscript and/or the Supplementary Materials. The raw data and reconstructed projections can be accessed at 10.16907/0b2214f9-750f-4592-a010-606822bff863.

## Code availability

The code and scripts developed and used for this study can be accessed at the following address: https://www.psi.ch/en/sls/csaxs/software, or obtained from the corresponding authors upon request.
