# Supplementary material for: In situ ptychographic nanotomography captures activation, mobility, and deactivation of supported catalysts
Source: Nat Commun. 2026 May 28;17:6917. doi: 10.1038/s41467-026-73365-w (PMC13389097; doi:10.1038/s41467-026-73365-w)
Supplement: Supplementary file 1 — Supplementary Information [file 41467_2026_73365_MOESM1_ESM.pdf]

Supplementary Information

In-Situ Ptychographic Nanotomography Captures Activation, Mobility, and Deactivation of Supported Catalysts

Arik Beck,*[1,2] Mirko Holler, *[3] Tomas Aidukas,[3] Andreas Menzel,[3] Manuel Guizar-Sicairos,[3,4] Jeroen A. van Bokhoven,[1,3] Johannes Ihli*[3,5]

Affiliations:

[1] ETH Zürich, 8093 Zürich, Switzerland

[2] Karlsruhe Institute of Technology KIT, 76187 Karlsruhe, Germany

[3] Paul Scherrer Institut, 5232 Villigen PSI, Switzerland

[4] Institute of Physics, École Polytechnique Fédérale de Lausanne (EPFL), 1015 Lausanne, Switzerland

[5] ALBA Synchrotron, 08290 Barcelona, Spain

*Correspondence and requests for materials should be addressed to arik.beck@kit.edu, mirko.holler@psi.ch or j.ihli@cells.es.

The Supplementary Information contains:

Figure S1 to S13

Table S1

Movie Captions S1-S4

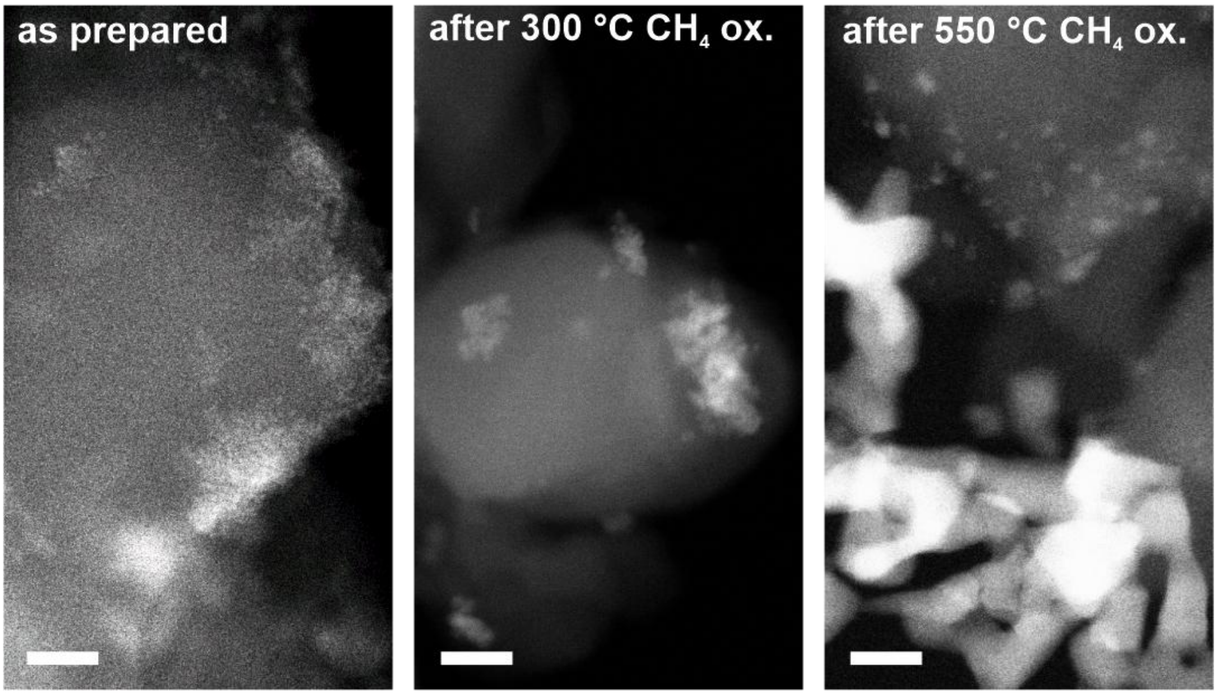

Supplementary Figure S1. Ex-Situ Scanning Transmission Electron Micrographs of the Supported Catalyst. Micrographs show the growth of the palladium particles as a function of temperature. Shown from left to right are images of the as-prepared catalyst, after heat treatment at 300 °C and 550 °C. Scale bars are 10 nm. Micrographs were acquired from mechanically fractured pieces of the supported catalyst.

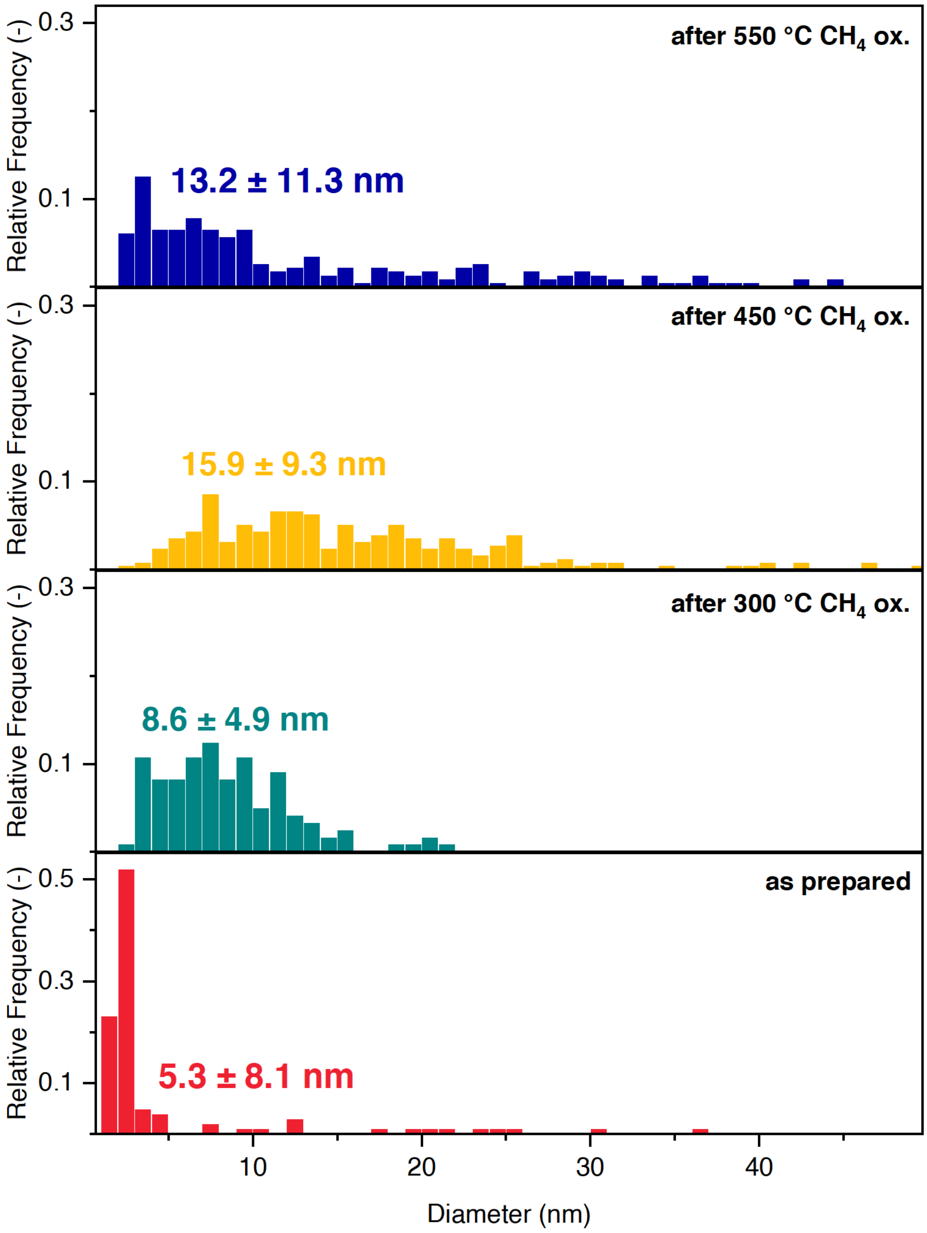

Supplementary Figure S2. Ex-Situ Scanning Transmission Electron Microscopy Derived Palladium Particle Size Distributions. Plotted are the PdO particle size distributions as found in the as-prepared catalyst and after CH_4 oxidation at 300 °C, 450 °C, and 550 °C. Reported is the Feret diameter determined using elliptical shapes. More than 200 particles were measured per condition.

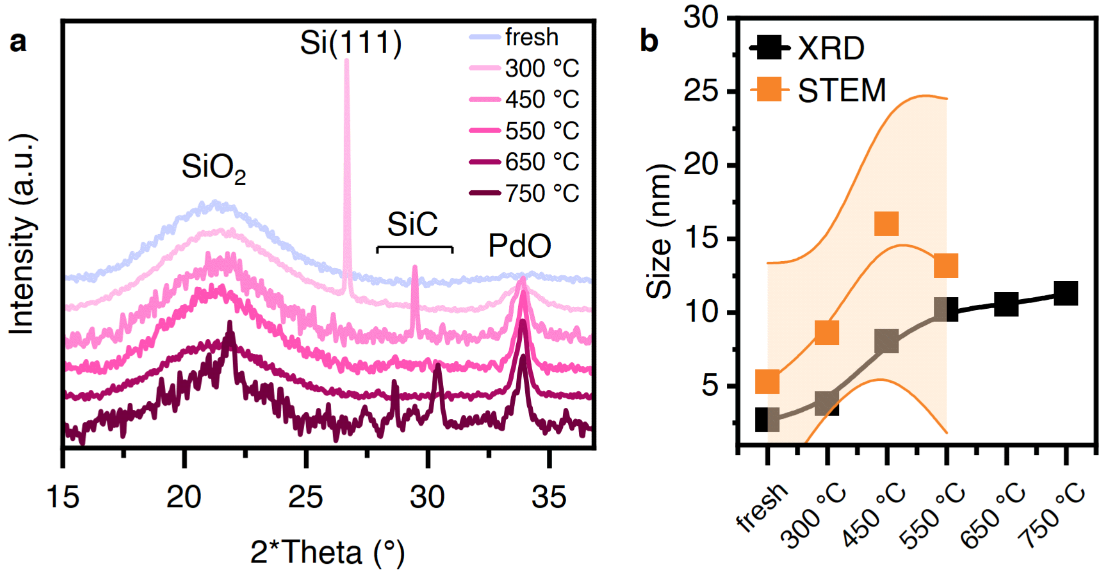

Supplementary Figure S3. Powder X-ray Diffraction of the Supported Catalyst as a Function of Temperature. (a) PXRD patterns of the as-prepared (fresh) catalyst and after CH_4 oxidation at 300 °C, 450 °C, 550 °C, 650 °C and 750 °C. Prominent reflections can be assigned to SiO_2, PdO, Si and SiC. No reflection of metallic Pd was detectable. The Si(111) reflection is owed to an incomplete coverage of the PXRD sample holder.

The detection of SiC stems from the presence of SiC in the powdered sample, a leftover from the catalytic bed dilution after the catalytic test. (b) Plotted are the Scherrer equation (black) and STEM (orange) derived PdO particle diameter as a function of temperature. The orange shaded area in (b) represents the standard deviation of the STEM particle size analysis (Figure S2).

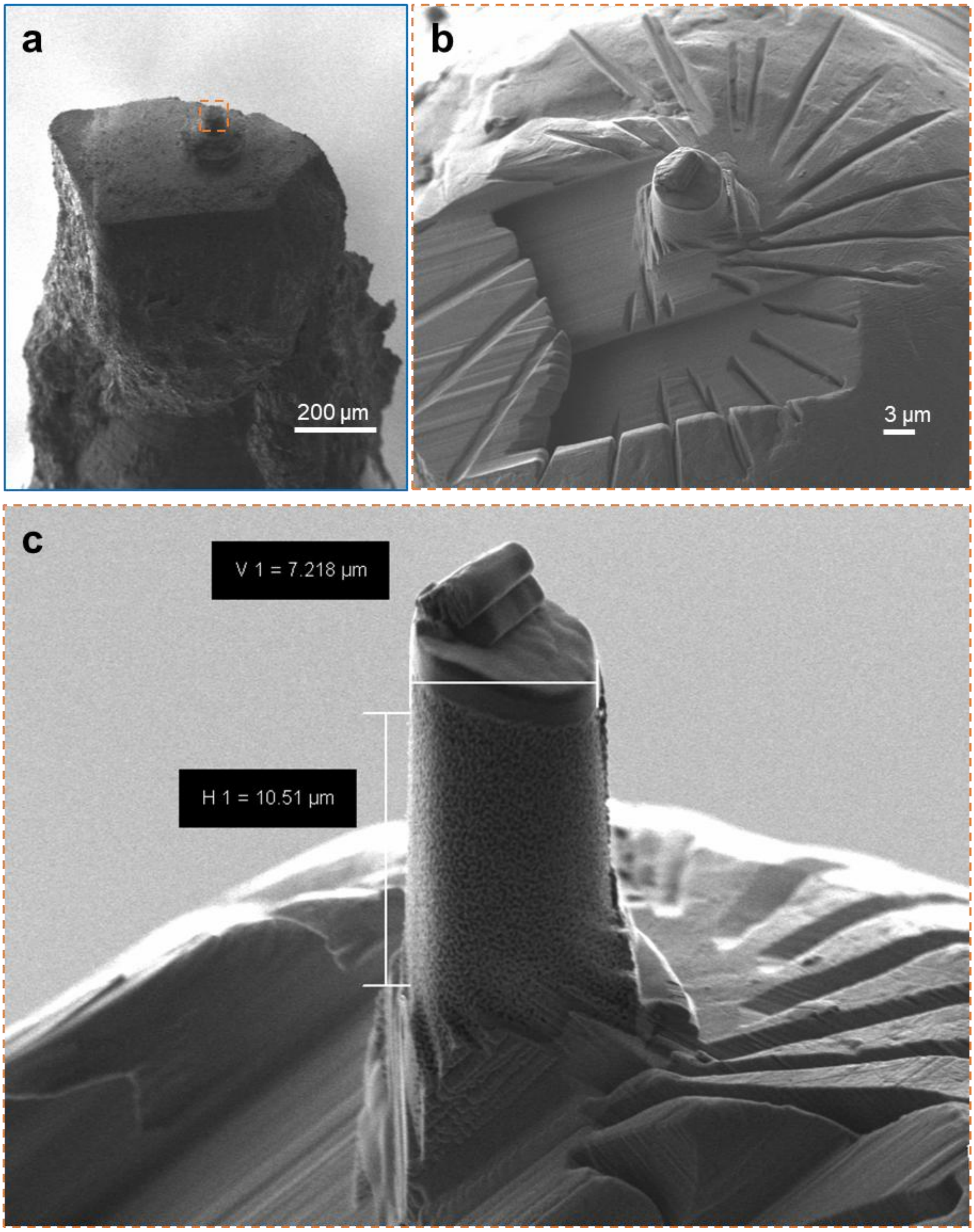

Supplementary Figure S4: Scanning Electron Micrographs of a Nanoporous Silica Sample Pillar. (a) Overview micrograph showing the sample after mounting of the glass bead on the tomography pin and following a pre-cut using a microlathe. (b&c) The final sample pillar after FIB milling of the pre-cut. Shown are a top-down and a side-view of the pillar. To note, are the exterior accessible or open pore structure, allowing for liquid infiltration, and the ion beam irradiation protective sample cap.

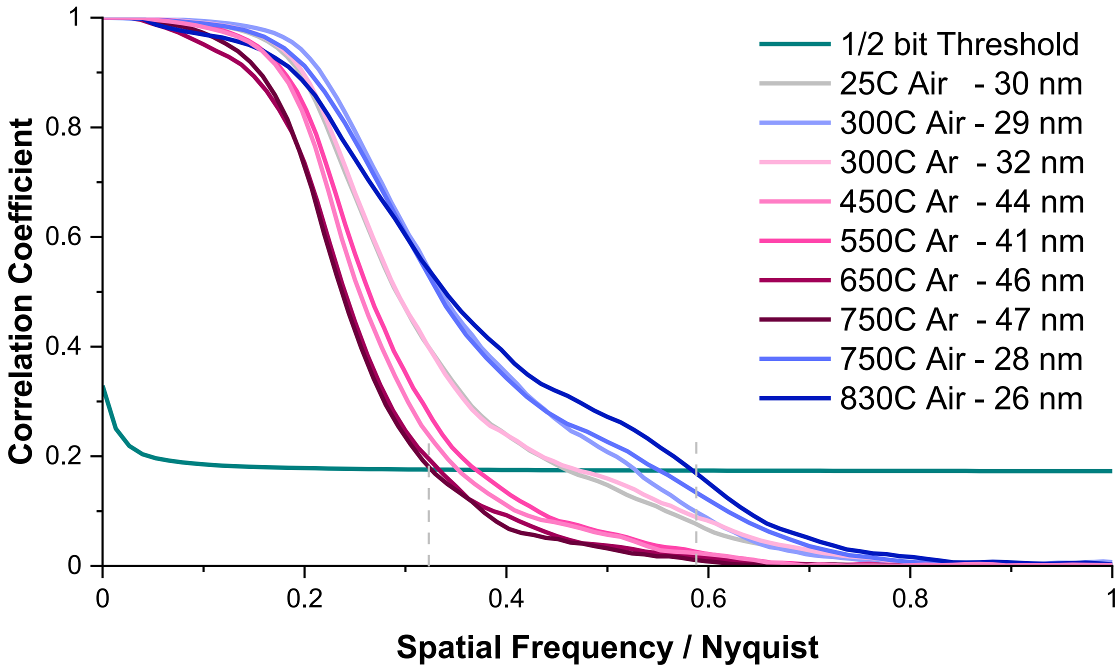

Supplementary Figure S5: Fourier Shell Correlation (FSC) Curves. Provided are FSC curves of the acquired phase / electron density tomograms. The *sample-average* half-period spatial resolution estimate is determined by the intersection of the FSC curve with the half-bit threshold curve (turquoise curve). The voxel size is (15.48 nm)^3. Variations in the FSC-derived spatial resolution estimates are currently primarily attributed to changes in the detectable evolution of the nanoparticles, rather than to instrumental instabilities. In particular, variations in particle size and dispersion of high-Z phases can strongly influence the amount of correlated high-frequency information contributing to the FSC. At room temperature up to approximately 400 °C, we find many well-dispersed and small metal precursor and Pd nanoparticles. This high number density of small, high-contrast scatterers provides abundant and repeatable high-frequency signal, resulting in comparatively high FSC values. Upon heating under inert (Ar) conditions, increased particle mobility promotes coarsening and agglomeration. As the number of small particles decreases and characteristic feature sizes increase, the amount of correlated high-frequency information is reduced, leading to a progressive degradation of the FSC with temperature. In contrast, at higher temperatures under oxidizing conditions (air), the system undergoes a qualitatively different transformation. Oxidation promotes the formation or condensation of dense, well-defined PdO particles from previously porous, fragmented metal domains. Many of these features were either below the detection threshold or contributed weakly and inconsistently to the Fourier correlations at earlier stages. Once transformed into stable, higher-density PdO particles, they generate stronger and more reproducible high-frequency contrast, which increases the correlated Fourier content and leads to an apparent recovery of the FSC. The 2D spatial resolution of ptychographic phase reconstructions, according to Fourier ring correlation (FRC) is 15 nm at 25˚C. No FRC tests were performed at elevated temperatures.

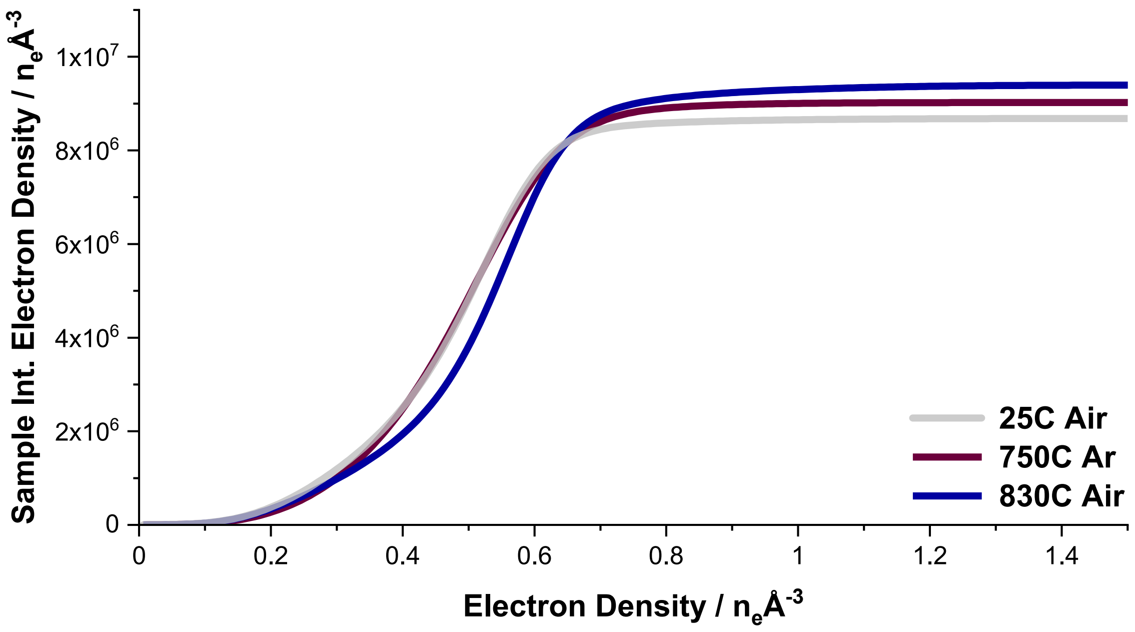

Supplementary Figure S6: Total Sample Mass as a Function of Environmental Conditions. Shown are cumulative or integrated electron density histograms of the supported catalyst tomograms acquired at 25ºC in air, at 750 ºC in an argon/ CH_4 atmosphere and at 830ºC in air. Analysed are identical sample volumes, i.e. containing the same number of voxels, with identical binning for histogram construction. The change in atmosphere density (air vs argon) was accounted for during tomogram reconstruction. Observable is an increase in integrated sample electron density of ~7% over the course of the measurement. Currently we assume this increase in density and thus total sample mass to be a result of (1) the formation of PdO, i.e. the uptake of oxygen and (2) the elimination of oxygen vacancies in the amorphous silica support (Figure S3).

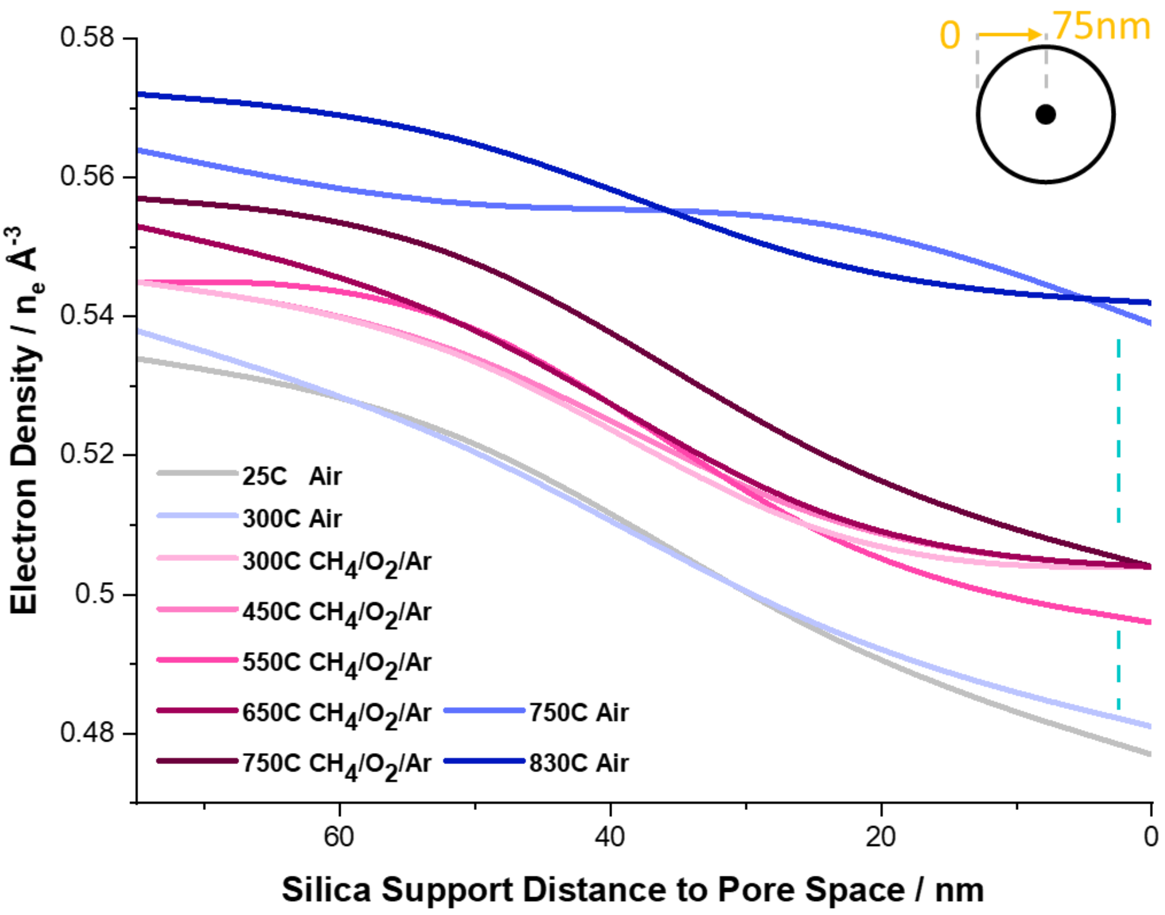

Supplementary Figure S7: Changes of the Silica Support. Plotted are electron density changes of the silica support as a function of environmental conditions and as a function of distance to the pore space. Voxels at 0 nm to the pore space are suggested to be in direct contact. Voxel at a distance of > 60 nm are assumed to represent the "bulk state". Excluded from the analysis were palladium-rich voxel (electron density > 0.8 n_e Å^{-3}). Evident is an increase in the support's electron density with increasing temperature. This increase is possibly due to the elimination of oxygen vacancies form the support and or the migration of Pd atoms into the support.[33,42] Notably, there is an initial spatial gradient in electron density, with voxel closer to the pore space possessing a lower electron density. While the existence of such a gradient is expected and owed to the supports synthesis route and partial volume effects, i.e. limited spatial resolution. The strong decay of this gradient with increasing temperature and change is atmosphere is surprising, and currently suggested to results from a combination of surface reconstruction, more efficient oxygen uptake at the support surface.

The here achieved spatial resolution and dimensionality prohibits us to investigate possible metal-support interactions,[51] i.e. accurately probe density and chemical changes of individual palladium particles and the surrounding support matrix. We hope to be able to probe these changes in a follow-up study utilizing recently developed sparse-hyper-dimensional ptychographic tomography approaches.[22]

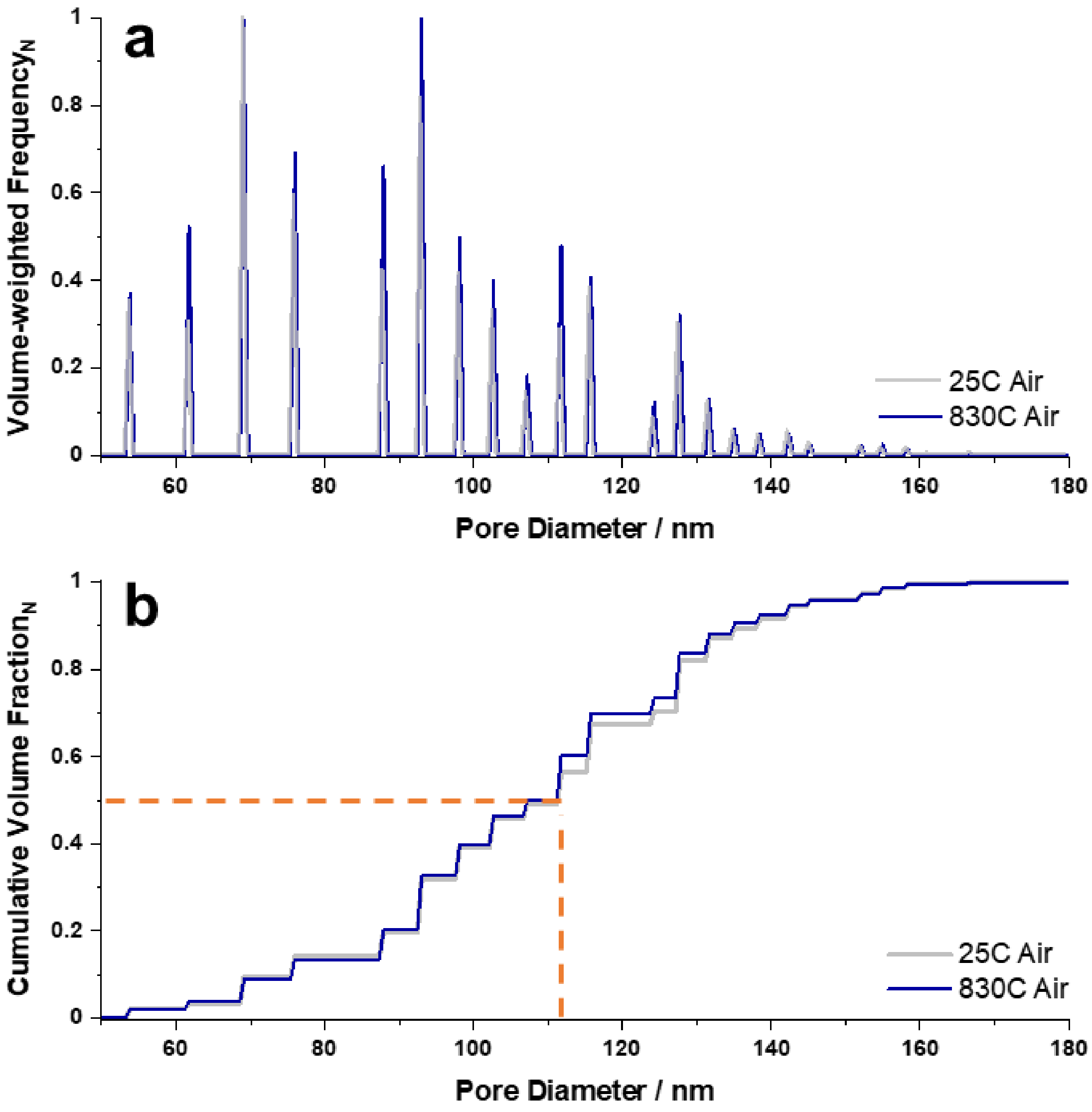

Supplementary Figure S8: Tomography Derived Pore Size Distributions. Shown are volume-weighted pore diameter distributions of the imaged catalyst pillar. For ease of visibility, we limit the display to the pore diameter distributions extracted from the tomograms acquired at 25˚C and 830˚C, i.e. the measured extremes. Provided are the pore diameter distributions (a) and the corresponding cumulative pore volume distributions (b).

The sample average, volume-weighted, pore diameter varies between 112 nm (25°C) and 107 nm (750°C) across the tested conditions, i.e. well in agreement with the literature reported and physisorption based diameter of 139 nm. Equally, the specific surface area of the support, assuming a fixed sample density of 2.20 g cm^{-3},[25] was calculated to be around 33 m^2g^{-1} across all environmental conditions. This is again well in agreement with the bulk measurement determined value. These observations suggest (1) the examined sample volume to be system representative and to be well describable via in-situ PXCT, (2) the support to be *dominantly* static across the tested environmental conditions (at the achieved spatial resolution) and (3) the observed local changes are ascribable to either Pd nanoparticle formation/ migration and Pd – support interactions.

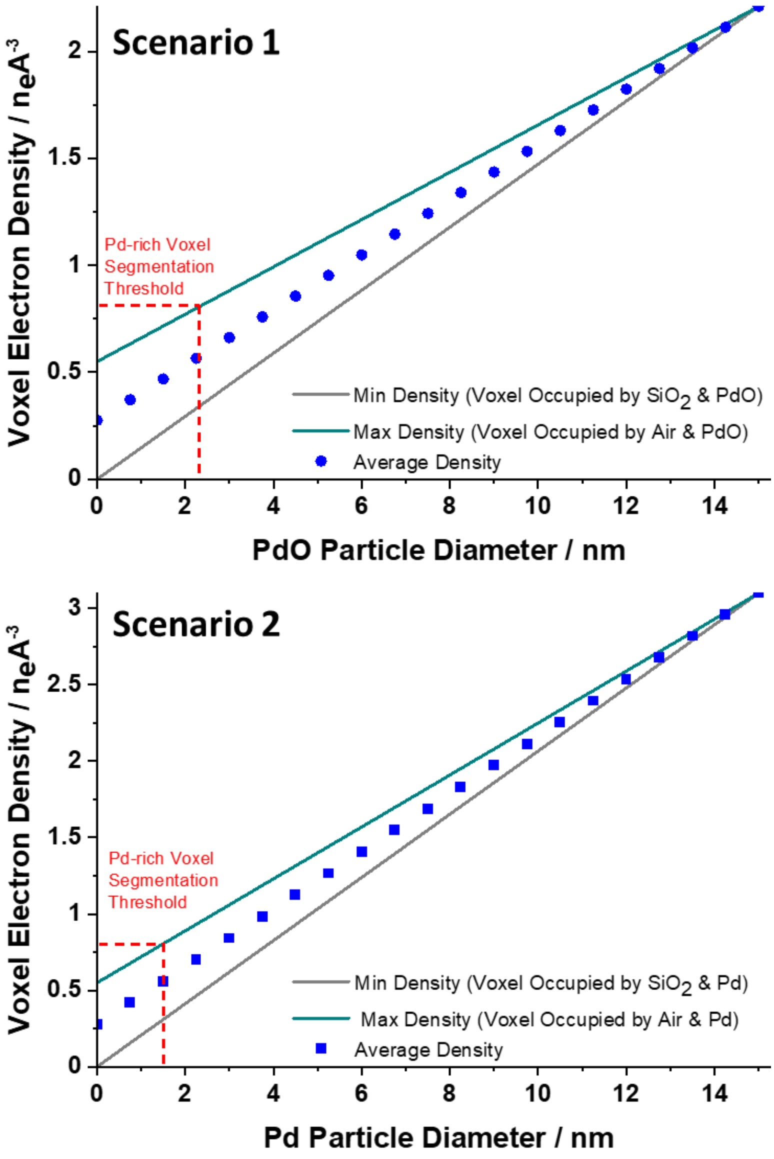

Supplementary Figure S9: Conversion of Electron Density to Palladium Particle Size. Shown is the correlation between a voxel`s electron density and palladium particle size. Plotted is the correlation for two scenarios. *Scenario 1* considers the sole presence of PdO nanoparticles in the sample. *Scenario 2* considers the sole presence of metallic Pd nanoparticles in the sample. The Pd or PdO particle size in a voxel can be estimated considering partial volume effects. Partial volume effects refer to the occupation of a voxel by a combination of materials (air, silica support, Pd or PdO) resulting in a volume-weighted - fractional occupancy - averaged electron density. To estimate the size of a (volumetrically equivalent) single Pd particle in a voxel, we can following consider the material combinations that lead to the minimum (--) or maximum (--) palladium particle size for a given electron density. The main text reported particle size estimates are based on the numerical average of both extremes (■) according to ***Scenario 1***. Scenario 1 was considered to result in more representative size estimates as PXRD and electron microscopy experiments suggest that the particles are PdO across the tested environmental conditions.

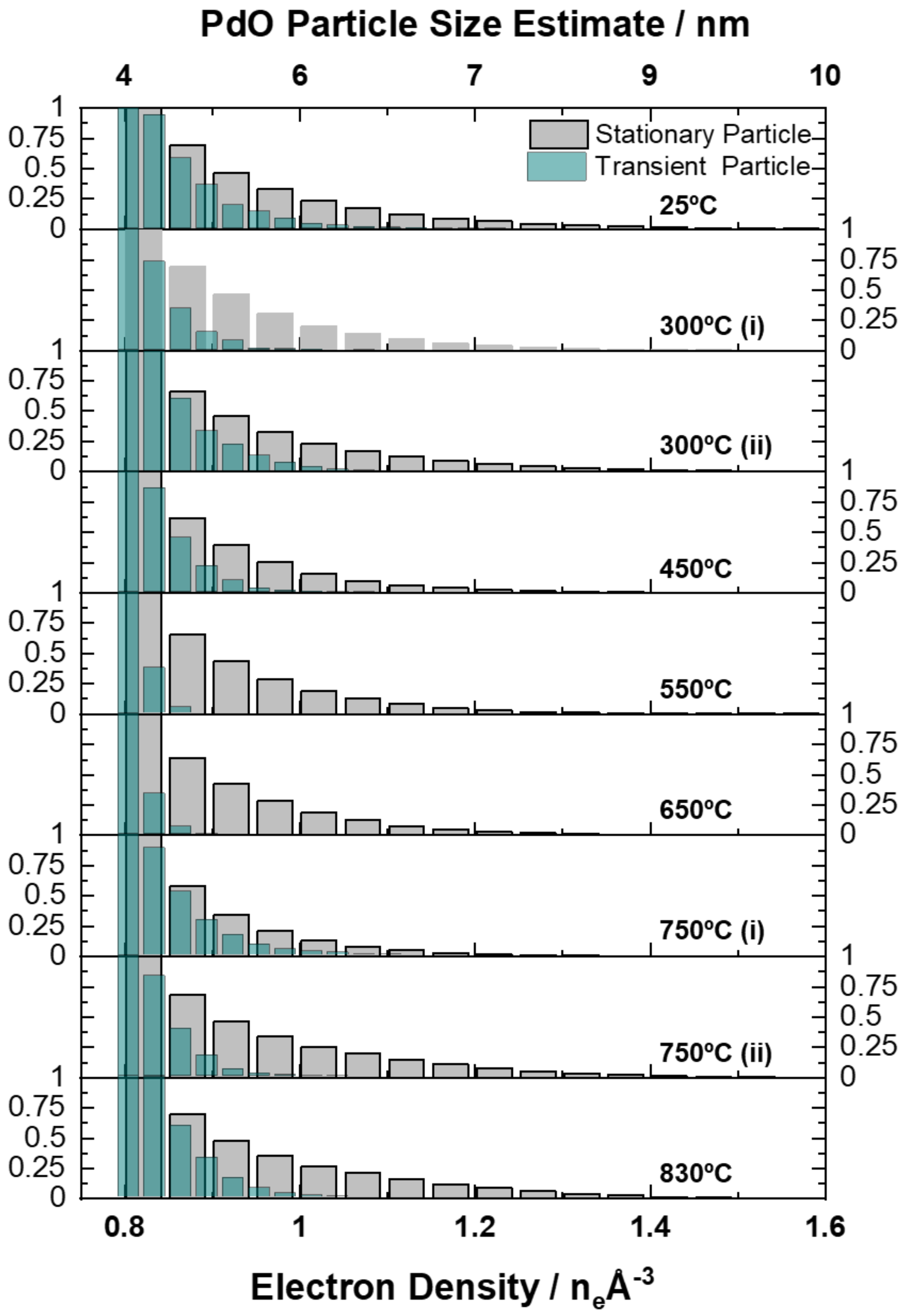

Supplementary Figure S10: Stationary and Mobile Palladium Particles. (a) Normalized electron density histograms of Pd-rich or particle carrying voxel as a function of temperature and atmosphere. A distinction is made between stationary and mobile palladium particles. Voxel *constantly* possessing an electron density greater than 0.80 n_e Å^{-3} starting from the first temperature where this threshold was crossed are classified as stationary or permanently occupied by a palladium particle. The remaining voxel are classified as transiently occupied.

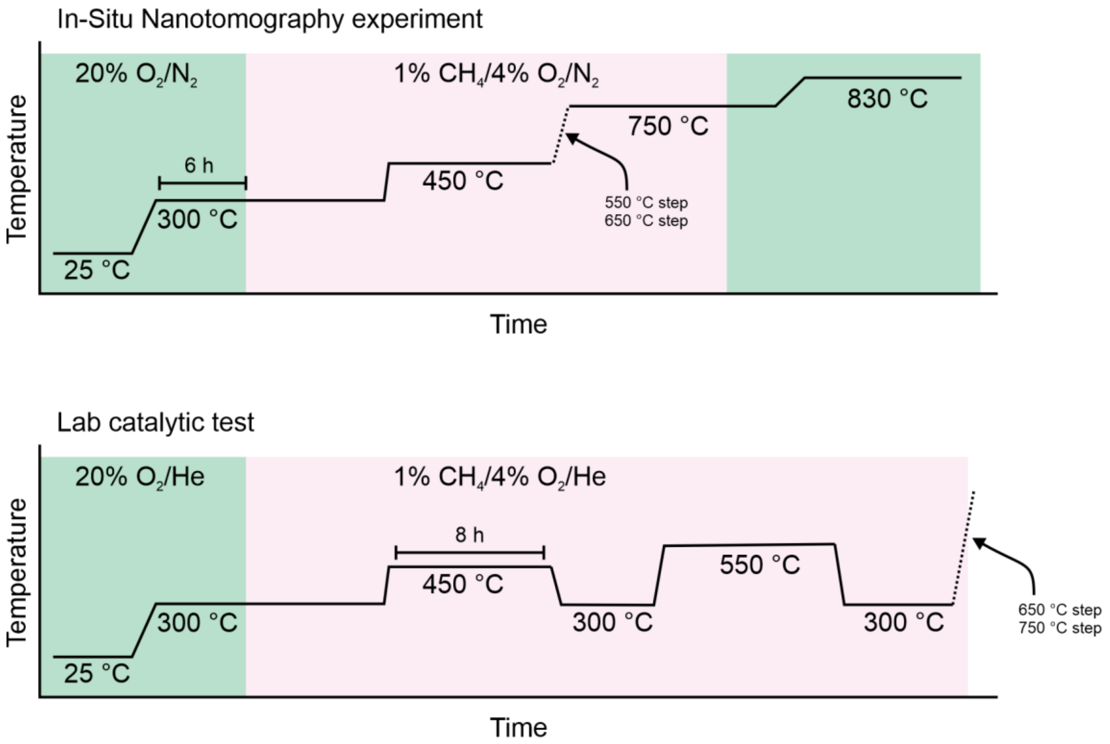

Supplementary Figure S11: Experimental Protocol. Scheme of the experimental protocols for the in-situ nanotomography experiment and the catalytic testing in the flow reactor. X and Y scales do not represent actual values but were adapted to increase readability of the scheme.

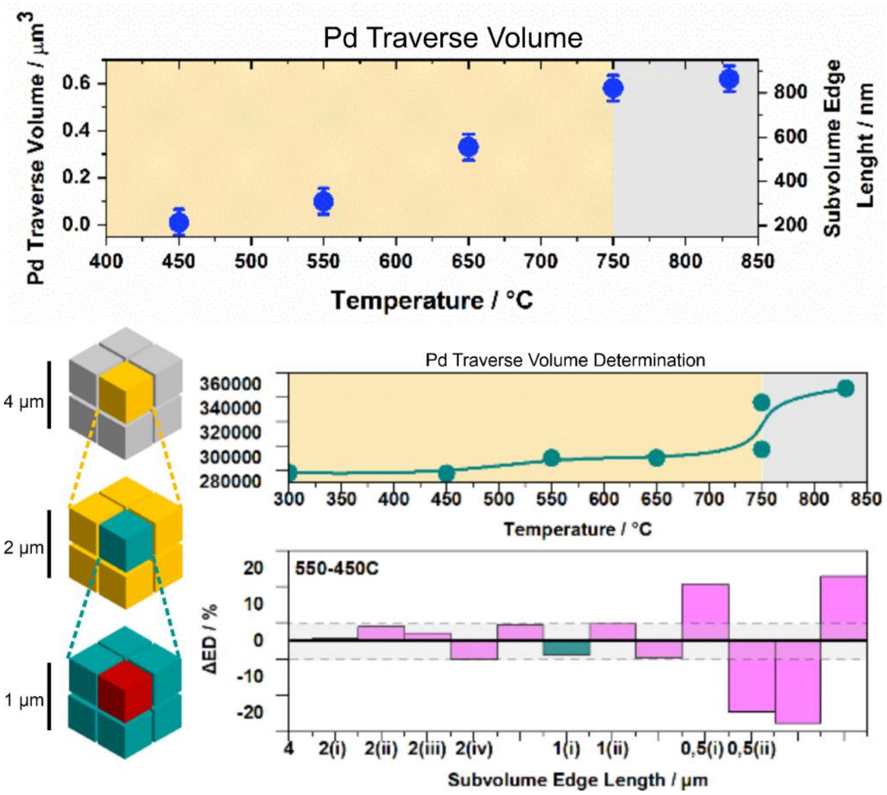

Supplementary Figure S12: Determination of the Pd Traverse Volume Top- palladium mass transport length as a function of temperature and atmosphere. Bottom – illustration depicting the determination process. (1) A central volume was iteratively sub-sampled into progressively smaller sub-volumes. (2) For each sub-volume, the integrated electron density change (ΔED) across a defined temperature window was then calculated. (3) The maximum Pd displacement length is inferred from the smallest sub-volume size at which a ≥5% deviation in normalized ΔED appears, indicating non-local Pd redistribution. Error bar represents the standard deviation.

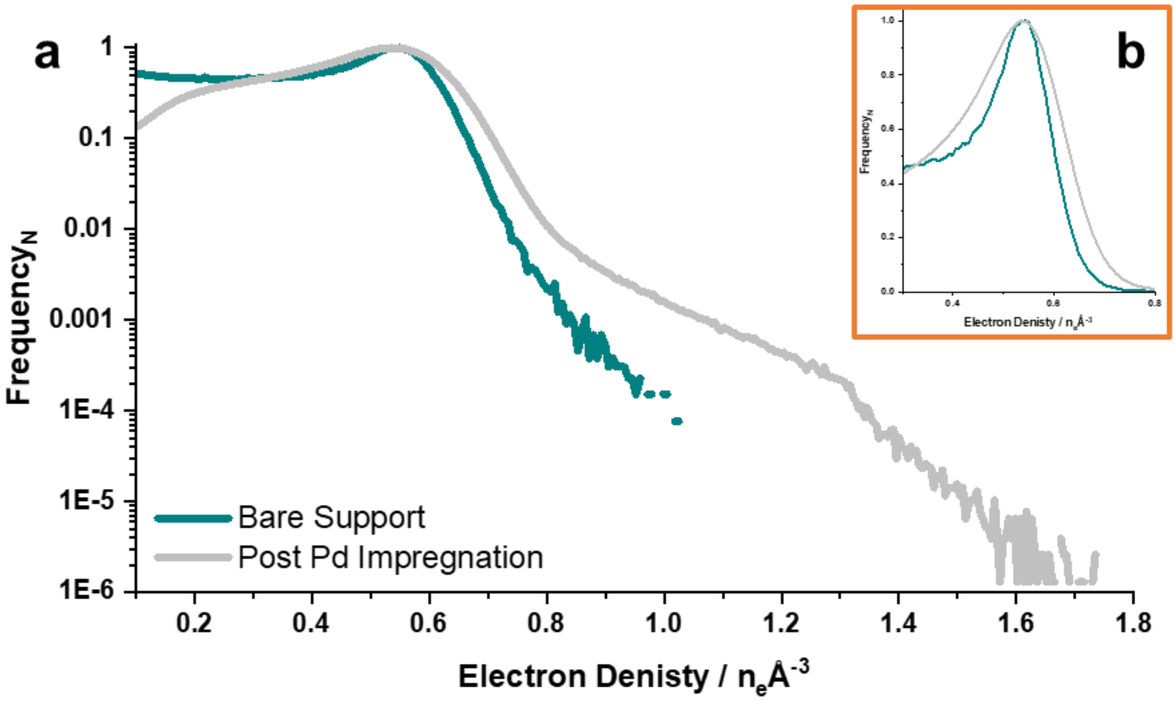

Supplementary Figure S13: Effect of the Wet Impregnation Process on the Silica Support. Electron density histograms derived from phase tomograms of the silica support, before (turquoise) and after (grey) the wet impregnation process. Histograms are show in (a) logarithmic and (b) linear scale. Both tomograms were acquired under identical conditions (25 °C, under air flow). Evident is that the silica peak position remains unchanged, suggesting that the bulk of the silica is unaffected by the impregnation process. However, (1) the impregnation process already results in a small population of Pd nanoparticles, albeit smaller than the achieved spatial resolution, evidenced by the high-density values (>1 n_e Å^{-3}). The electron density of the deposited palladium nitride is 0.98 n_e Å^{-3}. (2) The silica peak post impregnation process is significantly broadened. While the increased densities on the right flank can be explained by Pd particles and Pd nitride deposition on the supports surface, the changes on the left flank of the peak are likely due to a fraction of the pore space being occupied by precursor residues of the impregnation process.

**Compound**	**Electron Density @ 20˚C**
	**n_e Å^{-3}**
Air / **Argon / Pores**	~0.00
**Pd**	3.10
**PdO**	2.21
**$Pd(NO_2)_2$**	0.97
**SiO_2 (Amorphous)**	0.55
**Coke/ Organic Residues**	0.19-031

Supplementary Table S1: Electron Densities of known and Possible Supported Catalyst Components. The electron density of likely catalyst components was calculated using tabulated molecular weight and mass densities (Pd, PdO, gases) or extracted from the literature (SiO_2, Coke).[20,74] Temperature-dependent density variations were neglected, as the thermal expansion of relevant components (Pd, PdO, SiO_2) is estimated to affect electron density by less than the spatial resolution (~2% volume expansion across the full thermal range). Considering a sample diameter of 7 µm and assuming a solid body of silica we should be able to observe the pillar to expand by <50 nm as the temperature is raised to 830ºC. We don't observe such an expansion, likely due to the pillars nanoporous and the support`s amorphous character, i.e. most of the volume expansion will translate to increased strain levels and local displacements below the spatial resolution level.
