# Supplementary material for: In situ ptychographic nanotomography captures activation, mobility, and deactivation of supported catalysts
Source: Nat Commun. 2026 May 28;17:6917. doi: 10.1038/s41467-026-73365-w (PMC13389097; doi:10.1038/s41467-026-73365-w)
Supplement: Supplementary file 2 — Description of Additional Supplementary Files [file 41467_2026_73365_MOESM2_ESM.pdf]

Description of Additional Supplementary Files

File name: Supplementary Movie 1

Description: Volume Renderings of the Ptychographic Tomograms. Shown are animated volume renderings and cuts through the silica support as well as volume renderings that depict the location of voxels carrying palladium particles. Specifically, shown is the increase and decrease in palladium as inferred from local changes in electron density as a function of temperature and atmosphere. Changes in atmosphere at 300°C and 750°C and the subsequent acquisition of another tomograms at these temperatures are denoted (i & ii) in the movie. The display range for changes in palladium carrying voxel was limited to ± 1 n_e $Å^{-3}$ for ease of visualisation. The maximum observed changes, although very infrequent, were on the order of ± 2.1 n_e $Å^{-3}$ (Table S1)

File name: Supplementary Movie 2

Description: Volume Renderings of the Ptychographic Tomograms. Shown are animated volume renderings and cuts through the silica support as well as volume renderings that depict the location of voxels carrying palladium particles. Specifically, shown is the increase and decrease in palladium as inferred from local changes in electron density as a function of temperature and atmosphere. Changes in atmosphere at 300°C and 750°C and the subsequent acquisition of another tomograms at these temperatures are denoted (i & ii) in the movie. The display range for changes in palladium carrying voxel was limited to ± 1 n_e $Å^{-3}$ for ease of visualisation. The maximum observed changes, although very infrequent, were on the order of ± 2.1 n_e $Å^{-3}$ (Table S1)

File name: Supplementary Movie 3

Description: Stationary and Transient Palladium Particles. Provided is an animated volume rendering that shows the location of voxels that either carry stationary particle(s) (violet) or were temporarily occupied by a palladium particle (blue) over the course of the experiment.

File name: Supplementary Movie 4

Description: Stationary and Transient Palladium Particles. Provided is an animated volume rendering that shows the location of voxels that either carry stationary particle(s) (violet) or were temporarily occupied by a palladium particle (blue) over the course of the experiment.
