# Supplementary material for: In situ ptychographic nanotomography captures activation, mobility, and deactivation of supported catalysts
Source: Nat Commun. 2026 May 28;17:6917. doi: 10.1038/s41467-026-73365-w (PMC13389097; doi:10.1038/s41467-026-73365-w)
Supplement: Supplementary file 7 — Transparent Peer Review file [file 41467_2026_73365_MOESM7_ESM.pdf]

nature portfolio

Peer Review File

In-Situ Ptychographic Nanotomography Captures Activation, Mobility, and Deactivation of Supported Catalysts

Corresponding Author: Dr Arik Beck

This file contains all reviewer reports in order by version, followed by all author rebuttals in order by version.

Attachments originally included by the reviewers as part of their assessment can be found at the end of this file.

Version 0:

Reviewer comments:

Reviewer #1

(Remarks to the Author)
The paper presents an original, very timely, and important milestone for both Catalysis research and X-ray analysis, opening up a new avenue in functional studies of hard condensed matter. This work holds high significance in various fields, including, but not limited to, Chemistry, catalysis, and materials science. Performing functional studies using X-ray ptychographic tomography has never been reported previously. The work is performed on a high level of quality and is scientifically sound; however, several flaws were discovered and are attached in a separate file.

Reviewer #2

(Remarks to the Author)
This manuscript presents an excellent and pioneering demonstration of in-situ ptychographic nanotomography (PXCT) applied to a supported heterogeneous catalyst system. The study convincingly visualizes and quantifies the structural evolution and mobility of palladium nanoparticles throughout the entire catalytic lifecycle—activation, operation, and deactivation—under realistic geometries and controlled gas environments. By tracking over 100,000 particles within a hierarchically porous support, the authors reveal not only classical coarsening phenomena such as Ostwald ripening and particle migration/coalescence (PMC), but also a distinct long-range redistribution of Pd species over several hundred nanometers—an observation that significantly advances our mechanistic understanding of catalyst deactivation.
The experimental implementation is technically outstanding. The integration of a PXCT-compatible environmental control system with high-stability tomographic data acquisition is particularly impressive, enabling reliable long-duration imaging at a spatial resolution of approximately 35 nm. The data analysis, including voxel-based electron density evaluation, histogram evolution tracking, and migration velocity estimation via sub-volume sampling, is rigorous and methodologically sound. These approaches establish a quantitative framework for studying nanoscale dynamics within realistic catalyst architectures. Importantly, the results demonstrate that catalyst deactivation is driven not only by localized sintering but also by atmosphere-dependent, redox-mediated nanoparticle mobility. This discovery provides valuable insight into how gas environment and support interactions govern nanoparticle transport and stability, with direct implications for rational catalyst design.
From the perspective of PXCT methodology, this work represents a significant advance. It demonstrates the power of in-situ coherent diffraction imaging for quantitative three-dimensional observation of structural transformations under operando conditions. The study effectively bridges the resolution gap between high-resolution TEM observations and ensemble-averaged X-ray techniques, establishing PXCT as a practical and statistically meaningful tool for realistic catalyst analysis. The manuscript is scientifically solid, methodologically robust, and very well written. The data fully support the conclusions. I find no weakness that requires revision, and I strongly recommend publication in Nature Communications as it stands.

Reviewer #3

(Remarks to the Author)
This study applies in-situ ptychographic X-ray computed nanotomography (PXCT) with the aim of resolving catalyst restructuring under realistic operating conditions. While the authors visualize methane oxidation over Pd/SiO_2 and report two

apparent deactivation pathways—short-range ripening and long-range particle migration—the conclusions do not yet demonstrate a clear advancement in mechanistic insight beyond what is already well established in the catalyst sintering literature. The observed features, including particle migration over hundreds of nanometres, align with reported behaviours documented using higher-resolution microscopy techniques and therefore do not substantively extend current understanding.

First, in comparison with other characterisation technologies, the PXCT capabilities demonstrated here are not competitive in resolving the fundamental mechanisms of catalyst sintering. Short-range and long-range migration phenomena have been reported for decades using in-situ STM, TEM, and other imaging methods with atomic-scale resolution. Due to PXCT's spatial resolution limits in this study, the authors are unable to distinguish between key sintering mechanisms such as Smoluchowski ripening versus Ostwald ripening—mechanistic distinctions that have been previously clarified using atomic-resolution techniques. As a result, the study does not yet demonstrate unique or superior functionality of PXCT for sintering analysis.

Second, the authors aim to extrapolate the findings to general principles of heterogeneous catalyst sintering. However, methane catalytic combustion is a specialised, oxygen-rich oxidation reaction with distinct kinetic and thermodynamic characteristics, and therefore does not serve as a broadly representative model for heterogeneous catalysis. Third, the catalyst chosen for the study, Pd/SiO_2, is not a widely used formulation for methane combustion; continues reaction systems rely on Pd/Al_2O_3, Pd/CeO_2, or other supports that offer stronger metal–support interactions and markedly different restructuring behaviour. This limits the broader applicability of the conclusions.

Finally, the spatial resolution of in-situ PXCT (scale bar of 1 μm in this research) is not sufficient to capture the atomic to sub-nanometre–to–tens-of-nanometre structural changes where restructuring typically initiates and progresses at 200–500 °C. These early-stage processes, which govern catalyst lifetime and activity, remain inaccessible to the technique as implemented in this study. Collectively, these limitations constrain the extent to which the reported observations can be considered mechanistic discoveries or general principles of sintering behaviour in heterogeneous catalysts.

Version 1:

Reviewer comments:

Reviewer #1

(Remarks to the Author)
The authors have addressed all my points and I recommend the article for publication in the current state.

Open Access This Peer Review File is licensed under a Creative Commons Attribution 4.0 International License, which permits use, sharing, adaptation, distribution and reproduction in any medium or format, as long as you give appropriate credit to the original author(s) and the source, provide a link to the Creative Commons license, and indicate if changes were made.
In cases where reviewers are anonymous, credit should be given to 'Anonymous Referee' and the source.
The images or other third party material in this Peer Review File are included in the article's Creative Commons license,

unless indicated otherwise in a credit line to the material. If material is not included in the article's Creative Commons license and your intended use is not permitted by statutory regulation or exceeds the permitted use, you will need to obtain permission directly from the copyright holder.
To view a copy of this license, visit https://creativecommons.org/licenses/by/4.0/

Manuscript NCOMMS-25-84363- Response to Referees

We thank the referees and editorial staff for their detailed, constructive, and positive reviews of our manuscript, which have led to significant improvements.

As suggested, the requested missing technical information has been added. We corrected the manuscript section on the spatial resolution estimation in line with the reasoning of reviewer 2. In addition, in response to Reviewer 3, we have clarified the contributions of in-situ PXCT relative to high-resolution TEM and STM techniques. We have also specified the scope of our findings and highlighted the spatial resolution limits of our study.

Provided below is a point-by-point response to the referees' comments. The comments are presented in **black font**, the responses in **green font**. Changes to the manuscript are quoted below the respective replies. The revised manuscript is reproduced in **blue font** with yellow highlight for changed sections.

Reviewer #1 (Remarks to the Author):

The paper presents an original, very timely, and important milestone for both Catalysis research and X-ray analysis, opening up a new avenue in functional studies of hard condensed matter. This work holds high significance in various fields, including, but not limited to, Chemistry, catalysis, and materials science. Performing functional studies using X-ray ptychographic tomography has never been reported previously. The work is performed on a high level of quality and is scientifically sound; however, several flaws were discovered and are attached in a separate file.

1. Typo line 416: X-Xray.

→ Thank you for catching this typo. We have made the correction.

2. The authors don't provide information on the probe size used in PXCT methods, nor the scan time per projection (including the number of scanning points).

→ We thank the reviewer. The missing information has been added.

Changes to the manuscript:

Page 17, line 425: "PXCT[13,14] experiments were carried out 6.2 keV at the cSAXS beamline of the Swiss Light Source, Paul Scherrer Institut, Switzerland. The photon energy was selected using a double-crystal Si(111) monochromator. The horizontal aperture slits, located 22 m upstream of the sample was set to 20 μm in width, to create a horizontal virtual source point that coherently illuminated a Fresnel zone-plate, 200 μm in diameter, with an outermost zone width of 60 nm and a focal length of 60 mm. The Fresnel zone-plate was designed with locally displaced zones to improve imaging quality.[64] The sample was placed 1 mm downstream of the focus resulting in an illumination probe size of 4 um. Coherent diffraction patterns were acquired using an in-vacuum Eiger 1.5M area detector, with a 75 μm pixel size, placed 5.225 m downstream of the sample inside in an evacuated flight tube. "

Page 17, line 436: "The field of view of each projection, or ptychographic scan, varied from 10 x 5 μm^2 to 15 x 12.5 μm^2 (width x height). The sample was scanned using a Fermat-spiral scanning pattern with an average step size of 0.7 μm, resulting in around 350 scanning points per projection. The exposure time per scanning point was 0.05 seconds. Total acquisition time per projection including positing overhead was around 30 seconds. For each ptychographic scan or image reconstruction, a detector region of 900 × 900 pixels was used, resulting in a reconstructed image pixel size of 15.48 nm. Reconstructions were performed using the PtychoShelves package[67] with 500 iterations of the difference map algorithm[13] followed by 600 iterations of maximum likelihood refinement.[68] "

3. Why do FSCs get progressively worse for Ar conditions with the increase in temperature, and progressively get better (with increase of temperature) with Air? The maximal difference is up to 50%, and it is directly correlated with temperature and gas conditions. This behaviour also aligns with the confusion of interpretation of the result, which shows unexpected behaviour:

"Surprisingly, upon switching from the methane-rich reaction atmosphere to air at 750 °C we observe a marked collapse of the left-side tail of the silica peak in the histogram and a concurrent rise in palladium-rich voxels. We believe this change is caused by oxidation-driven reorganization of previously porous, fragmented, or partially reduced palladium particles into more densely packed and well-defined PdO particles."

Examining the list of FSC numbers across different temperatures and atmospheric types reveals a correlation between temperature and the FSC numbers. Until the atmosphere and temperature were changed, a very high jump happened right after the temperature was raised to 450 °C, and then FSC dropped back under the same temperature with a change of atmosphere:

While the authors attempted to explain the behavior by the influence of the atmosphere on laser interferometer precision, it does not align well with the fact that very close FSC numbers are exhibited by both atmospheres at identical temperatures (300 °C, Air and Ar). In my naive view, the sample underwent a more complex process, which is now unexplained. Moreover, authors found pores that are either close to the FSC estimated resolution or are no more than 2 times that resolution (e.g., Fig. S8), which can be directly affected by fluctuations in resolution—the main question is whether these fluctuations were related to actual changes in the sample. A more thorough analysis of this behaviour is vital for the proper interpretation of the obtained results.

We thank the reviewer for this important observation. After careful re-evaluation of the tomographic reconstructions together with the acquisition metadata, we agree that the observed atmosphere- and temperature-dependent trends in the FSC are more plausibly explained by changes in the sample, rather than by systematic variations in instrumental stability or interferometer performance.

In particular, the similarity of FSC values obtained under air and Ar at identical temperatures (e.g., 300 °C) argues against a dominant instrumental origin as the reviewer points out. Instead, the FSC behaviour can be consistently rationalized by considering how Pd particle size, dispersion, and density of high-Z phases evolve under different thermal and atmospheric conditions, and how these changes affect the correlated high-frequency signal in the reconstructions.

First, we can largely exclude the silica framework as the driver of the observed FSC trends. The amorphous SiO_2 matrix is a low-Z phase and contributes only weakly to high-frequency contrast compared to Pd- or PdO-rich regions. Consequently, changes in FSC at the spatial frequencies relevant here are dominated by the nanoparticles.

At room temperature up to approximately 400 °C, the metal precursor is finely dispersed, with many small, well-distributed nanoparticles. This high number density of small, high-contrast scatterers provides abundant and repeatable high-frequency signal, resulting in comparatively high FSC values. Upon heating under inert (Ar) conditions, increased particle mobility promotes coarsening and agglomeration. As the number of small particles decreases and characteristic feature sizes increase, the amount of correlated high-frequency information is reduced, leading to a progressive degradation of the FSC with temperature. In contrast, at higher temperatures under oxidizing conditions (air), the system undergoes a qualitatively different transformation. Oxidation promotes the formation or condensation of dense, well-defined PdO particles from previously porous, fragmented, or partially reduced metal domains. Many of these features were either below the detection threshold or contributed weakly and inconsistently to the Fourier correlations at earlier stages. Once transformed into stable, higher-density PdO particles, they generate stronger and more reproducible high-frequency contrast, which increases the correlated Fourier content and leads to a recovery (or improvement) of the FSC despite the elevated temperature.

In this sense, the FSC variations reflect the detectable change in population of high-contrast smaller nanoparticles rather than a monotonic dependence on temperature and atmosphere alone. We have revised the manuscript to clarify this as follows:

Page 32, line 747: "**Supplementary Figure S5: Fourier Shell Correlation (FSC) Curves.** Provided are FSC curves of the acquired phase / electron density tomograms. The *sample-average* half-period spatial resolution estimate is determined by the intersection of the FSC curve with the half-bit threshold curve (turquoise curve). The voxel size is $(15.48\ \text{nm})^3$. Variations in the FSC-derived spatial resolution estimates are currently primarily attributed to changes in the detectable evolution of the nanoparticles, rather than to instrumental instabilities. In particular, variations in particle size and dispersion of high-Z phases can strongly influence the amount of correlated high-frequency information contributing to the FSC. At room temperature up to approximately 400 °C, we find many well-dispersed and small metal precursor and Pd nanoparticles. This high number density of small, high-contrast scatterers provides abundant and repeatable high-frequency signal, resulting in comparatively high FSC values. Upon heating under inert (Ar) conditions, increased particle mobility promotes coarsening and agglomeration. As the number of small particles decreases and characteristic feature sizes increase, the amount of correlated high-frequency information is reduced, leading to a progressive degradation of the FSC with temperature. In contrast, at higher temperatures under oxidizing conditions (air), the system undergoes a qualitatively different transformation. Oxidation promotes the formation or condensation of dense, well-defined PdO particles from previously porous, fragmented metal domains. Many of these features were either below the detection threshold or contributed weakly and inconsistently to the Fourier correlations at earlier stages. Once transformed into stable, higher-density PdO particles, they generate stronger and more reproducible high-frequency contrast, which increases

the correlated Fourier content and leads to an apparent recovery of the FSC. The 2D spatial resolution of ptychographic phase reconstructions, according to Fourier ring correlation (FRC) is 15 nm at 25˚C. No FRC tests were performed at elevated temperatures. "

Overall, I consider this paper of high quality and can recommend it for publication after the authors address all the concerns listed.

Reviewer #2 (Remarks to the Author):

This manuscript presents an excellent and pioneering demonstration of in-situ ptychographic nanotomography (PXCT) applied to a supported heterogeneous catalyst system. The study convincingly visualizes and quantifies the structural evolution and mobility of palladium nanoparticles throughout the entire catalytic lifecycle—activation, operation, and deactivation—under realistic geometries and controlled gas environments. By tracking over 100,000 particles within a hierarchically porous support, the authors reveal not only classical coarsening phenomena such as Ostwald ripening and particle migration/coalescence (PMC), but also a distinct long-range redistribution of Pd species over several hundred nanometers—an observation that significantly advances our mechanistic understanding of catalyst deactivation.

The experimental implementation is technically outstanding. The integration of a PXCT-compatible environmental control system with high-stability tomographic data acquisition is particularly impressive, enabling reliable long-duration imaging at a spatial resolution of approximately 35 nm. The data analysis, including voxel-based electron density evaluation, histogram evolution tracking, and migration velocity estimation via sub-volume sampling, is rigorous and methodologically sound. These approaches establish a quantitative framework for studying nanoscale dynamics within realistic catalyst architectures.

Importantly, the results demonstrate that catalyst deactivation is driven not only by localized sintering but also by atmosphere-dependent, redox-mediated nanoparticle mobility. This discovery provides valuable insight into how gas environment and support interactions govern nanoparticle transport and stability, with direct implications for rational catalyst design.

From the perspective of PXCT methodology, this work represents a significant advance. It demonstrates the power of in-situ coherent diffraction imaging for quantitative three-dimensional observation of structural transformations under operando conditions. The study effectively bridges the resolution gap between high-resolution TEM observations and ensemble-averaged X-ray techniques, establishing PXCT as a practical and statistically meaningful tool for realistic catalyst analysis.

The manuscript is scientifically solid, methodologically robust, and very well written. The data fully support the conclusions.

I find no weakness that requires revision, and I strongly recommend publication in Nature Communications as it stands.

Response:

We thank the referee for the time and effort spent on the manuscript. We highly appreciate that the reviewer recognized the relevance and importance that our work carries to the community.

Reviewer #3 (Remarks to the Author):

This study applies in-situ ptychographic X-ray computed nanotomography (PXCT) with the aim of resolving catalyst restructuring under realistic operating conditions. While the authors visualize methane oxidation over Pd/SiO_2 and report two apparent deactivation pathways—short-range ripening and long-range particle migration—the conclusions do not yet demonstrate a clear advancement in mechanistic insight beyond what is already well established in the catalyst sintering literature. The observed features, including particle migration over hundreds of nanometres, align with reported behaviours documented using higher-resolution microscopy techniques and therefore do not substantively extend current understanding.

Response:

We thank the reviewer for the time taken to consider our work and for the comments, which have helped us to improve our manuscript. We have revised the manuscript introduction to better articulate how in-situ PXCT contributes to the broader understanding of catalyst restructuring and deactivation by providing non-destructive, three-dimensional in-situ information across multiple length scales that complement atomic-resolution studies. In line with recent perspectives (1.Unifying views on catalyst deactivation, 2. Spatial and temporal exploration of heterogeneous catalysts with synchrotron radiation) emphasizing the need for multiscale, multimodal and in-situ/ operando characterisation to fully explore heterogeneous catalysis, we now stress more explicitly that yet no single technique or length scale can provide a complete picture, and that our results are intended to be integrated with, rather than compete with transmission electron microscopy insights. We have further revised the manuscript to distinguish the unique non-destructive volumetric and transport insights provided by in-situ PXCT from those obtained via traditional high-resolution microscopy. Below we address the reviewer's specific points and outline the corresponding clarifications and manuscript revisions.

First, in comparison with other characterisation technologies, the PXCT capabilities demonstrated here are not competitive in resolving the fundamental mechanisms of catalyst sintering. Short-range and long-range migration phenomena have been reported for decades using in-situ STM, TEM, and other imaging methods with atomic-scale resolution. Due to PXCT's spatial resolution limits in this study, the authors are unable to distinguish between key sintering mechanisms such as Smoluchowski ripening versus Ostwald ripening—mechanistic distinctions that have been previously clarified using atomic-resolution techniques. As a result, the study does not yet demonstrate unique or superior functionality of PXCT for sintering analysis.

Response:

High-resolution characterisation techniques, such as TEM for powdered catalysts or STM for planar model systems, provide an atomic- to nanometre-scale view of sintering that remains unmatched by most spatially resolved methods, and have been indispensable for distinguishing microscopic pathways such as different ripening mechanisms. At the same time, these methods are typically constrained to sub-micron sample volumes, limited fields of view and observation times, and require destructive sample preparation, which makes it impossible to follow restructuring non-destructively within intact, use-representative catalyst bodies and across large particle populations.

In-situ PXCT is therefore not in competition with TEM or STM, but is intended to be complementary, providing a different perspective on catalyst behaviour. As stated in the conclusions of the manuscript, its role is to bridge or narrow the information gap between in-situ electron microscopy and the statistical relevance of averaging in-situ methods. It enables non-destructive, three-dimensional, quantitative imaging of restructuring within the intact pore network at tens-of-nanometre resolution, allowing us to determine transport lengths, migration velocities and atmosphere-dependent redistribution for 100,000s of particles in a realistic catalyst geometry. Such volumetric and statistical information is impossible to obtain with atomic resolution techniques, yet is directly relevant for linking microscopic pathways to catalyst-body-scale deactivation and design, and thus represents a different way to understand catalyst behaviour.

The present work constitutes a first demonstration of in-situ PXCT applied to a heterogeneous catalyst and one if not the first demonstration of controlled in-situ PXCT, and we explicitly acknowledge in the manuscript that the technique is still developing and not yet as mature as TEM in terms of spatial and temporal resolution, i.e. improvements are expected. Nonetheless, the quantitative estimation of transport velocities and migration distances over hundreds of nanometres for a statistically significant particle population, within a use-representative volume, has not previously been reported and is directly relevant for the design of catalyst bodies and for constraining theoretical models of catalyst restructuring.

We have revised our manuscript to make the objectives and distinctions of our experiments to mechanistic findings obtained by other tools clearer to the reader.

Changes to the manuscript:

Page 3 Line 60: *"High-resolution imaging methods, in contrast, can provide these and allow for a more detailed understanding of catalyst behaviour, including the mobility of individual nanoparticles.*[9–11] *For example, using in-situ transmission electron microscopy (TEM), we showed that the nanoparticles in a supported catalyst can become highly mobile under reaction conditions, questioning a previously suggested pure and local Ostwald Ripening driven deactivation.*[12] *Such TEM studies are essential to resolve atomic- to nanometre-scale mechanisms of sintering and metal–support interaction.*[5] *However, transmission electron microscopy is constrained to sub-micron sample volumes*[5] *and commonly involves destructive sample preparation steps to obtain electron transparent specimen. This limits its use for studying realistic catalyst architectures and statistically representative particle behaviour. This limitation becomes increasingly important as the focus shifts towards a holistic description of industrial catalysts, their function and deactivation, emphasising the need for complementary, non-destructive approaches that probe structure and chemistry across representative sample volumes and over multiple length scales, thereby linking atomic-scale insights to mesoscale processes.*[4,13] *Ptychographic X-ray computed tomography (PXCT) is an emerging complementary investigative method,*[13–16] *allowing us to measure such larger and intact sample volumes with sufficient spatial resolution to identify the nanoparticles within a supported catalysts."*

Page 4, line 109: "However, ex-situ measurements do not reveal the processes that lead to the agglomeration, especially palladium transport process and their associated length scales and velocities."

Page 4, line 111: "To capture those transport processes without destroying the pore architecture of the catalysts, we then examined a 7 μm-wide catalyst pillar (Figure S4) via in-situ ptychographic X-ray computed tomography,[21,26] capturing the evolution of the catalyst from nitrate-rich precursor decomposition to deactivation (**Figure 1b**)."

Second, the authors aim to extrapolate the findings to general principles of heterogeneous catalyst sintering. However, methane catalytic combustion is a specialised, oxygen-rich oxidation reaction with distinct kinetic and thermodynamic characteristics, and therefore does not serve as a broadly representative model for heterogeneous catalysis. Third, the catalyst chosen for the study, Pd/SiO_2, is not a widely used formulation for methane combustion; continues reaction systems rely on Pd/Al_2O_3, Pd/CeO_2, or other supports that offer stronger metal–support interactions and markedly different restructuring behaviour. This limits the broader applicability of the conclusions.

Response:

We agree that studies conducted under specific conditions, such as oxygen-rich atmospheres and high temperatures, do not by themselves yield results that are directly generalisable to all forms of heterogeneous catalysis. However, this limitation is not unique to our work: no single set of testing conditions or model system can provide a universally descriptive model that covers the full diversity of catalytic materials and operating regimes. We apologise if our wording suggested otherwise. The demonstrated methodology is, however, general and can be applied to a wide range of material systems and environmental conditions, making it relevant to the broader heterogeneous catalysis, geological, structural and functional materials communities.

We have revised the manuscript text to specify more clearly the system and conditions investigated and to avoid overly generalising language.

Regarding the choice of catalyst system, our intent was not to study the most active or industrially optimised formulation for methane oxidation, but to employ a palladium catalyst supported on SiO_2, one of the most common oxide supports used in heterogeneous catalysis. This system provides a chemically simple and well-established platform for investigating restructuring and transport in a hierarchically porous support under severe oxidative conditions. Supported palladium on CeO_2 or Al_2O_3 are other options and are subject to future studies.

Below is a non-exhaustive list of relevant research articles investigating Pd/SiO_2 catalyst for methane oxidation.

- Fuel 103, 2013, 1032-1038
- Journal of Industrial and Engineering Chemistry 99, 2021, 90-97
- Applied Catalysis A: General 283, 2005, 225-233
- Applied Catalysis A: General 134, 1996, 203-215
- Ind. Eng. Chem. Res. 2021, 60, 20, 7545–7557

Changes to the manuscript:

Page 6, line 224: "In-situ ptychographic nanotomography enabled us to monitor the evolution of a supported Pd/SiO_2 catalyst for methane oxidation across a full activation–operation–deactivation cycle, from room temperature up to 830 °C under oxidizing and methane oxidation atmospheres."

Page 6, line 245: "These findings indicate two distinct transport and deactivation pathways for Pd on SiO_2 supports under oxygen rich conditions: (1) a short-range, surface-mediated coarsening process, e.g. a combination of Ostwald ripening and PCM, that governs initial nanoparticle formation and growth, and (2) a long-range particle redistribution mechanism that operates beyond the scale of conventional surface diffusion."

Page 7, line 281: "Accordingly, strategies that rely solely on optimizing interparticle distances or initial dispersion patterns[46,47] may prove insufficient to prevent deactivation under such conditions. Instead, more comprehensive approaches are needed: anchoring particles through tailored metal–support interactions [40,48,49] and designing support architectures that physically restrict mobility, e.g., via tortuosity engineering or controlled confinement may offer more robust stabilization under harsh conditions."

Page 8, line 300: "By monitoring the evolution of over 100,000 individual catalyst particles in a geometry representative of their actual usage and under conditions that approach their real-world application, we revealed that Pd/SiO_2 catalyst deactivation under methane oxidation and calcinations conditions of is governed not only by localized coarsening via ripening and coalescence but also by long-range redistribution of entire nanoparticles across hundreds of nanometers."

Finally, the spatial resolution of in-situ PXCT (scale bar of 1 µm in this research) is not sufficient to capture the atomic to sub-nanometre–to–tens-of-nanometre structural changes where restructuring typically initiates and progresses at 200–500 °C. These early-stage processes, which govern catalyst lifetime and activity, remain inaccessible to the technique as implemented in this study.

Collectively, these limitations constrain the extent to which the reported observations can be considered mechanistic discoveries or general principles of sintering behaviour in heterogeneous catalysts.

Response:

We would like to clarify that the spatial resolution of the in-situ PXCT measurements in this study is ~ 35 nm, with a voxel size of 15.5 nm. The 1 µm are scale bars in some figures, not resolution. The sample-average resolution estimate is explicitly stated at the beginning and derived from Fourier Shell Correlation analysis (Figure S5).

We fully agree that sub-nanometre and atomic-scale structural changes, where many sintering processes are initiated, are beyond the capabilities of the present implementation and currently remain the domain of high-resolution electron microscopy and related techniques. As pointed out in the manuscript our focus in this work is instead on structural changes at the tens-of-nanometre scale and on mesoscale transport / length scales that are directly resolved by our PXCT data and that are highly relevant for connecting local sintering mechanisms to catalyst-body-scale deactivation.

We further state in the discussion and outlook that technological advances potentially will bring us into the realm of directly visualising nanometre resolution. In our view, this would represent a true paradigm shift in in-situ characterisation of heterogeneous catalysts.

Page 7, line 286: "*While quantitative in-situ nanotomography facilitates a more use-representative and holistic characterisation of heterogeneous catalysts, the approach is currently bounded by spatial and temporal resolution as well as chemical specificity. These limitations currently prevent us from directly probing local metal-support interactions,*[25,50,51] *i.e. probe the morphology and chemistry of individual particles and the surrounding support, or the kinetics of migration events at the single-particle level.*"

As outlined in the results section of the manuscript, while bounded by spatial-resolution in term of direct visualisation, through the quantitative electron-density contrast, we are able to infer nanometre-scale changes from voxel-level density variations across environmental conditions, for example in terms of equivalent particle size and sintering behaviour, even when individual particles are not directly resolved.

1. Typo line 416: X-Xray.
2. The authors don't provide information on the probe size used in PXCT methods, nor the scan time per projection (including the number of scanning points).
3. Why do FSCs get progressively worse for Ar conditions with the increase in temperature, and progressively get better (with increase of temperature) with Air? The maximal difference is up to 50%, and it is directly correlated with temperature and gas conditions. This behaviour also aligns with the confusion of interpretation of the result, which shows unexpected behaviour:

   "Surprisingly, upon switching from the methane-rich reaction atmosphere to air at 750 °C we observe a marked collapse of the left-side tail of the silica peak in the histogram and a concurrent rise in palladium-rich voxels. We believe this change is caused by oxidation-driven reorganization of previously porous, fragmented, or partially reduced palladium particles into more densely packed and well-defined PdO particles."

   Examining the list of FSC numbers across different temperatures and atmospheric types reveals a correlation between temperature and the FSC numbers. Until the atmosphere and temperature were changed, a very high jump happened right after the temperature was raised to 450 °C, and then FSC dropped back under the same temperature with a change of atmosphere:

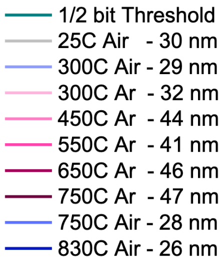

   While the authors attempted to explain the behavior by the influence of the atmosphere on laser interferometer precision, it does not align well with the fact that very close FSC numbers are exhibited by both atmospheres at identical temperatures (300 °C, Air and Ar). In my naive view, the sample underwent a more complex process, which is now unexplained. Moreover, authors found pores that are either close to the FSC estimated resolution or are no more than 2 times that resolution (e.g., Fig. S8), which can be directly affected by fluctuations in resolution—the main question is whether these fluctuations were related to actual changes in the sample. A more thorough analysis of this behaviour is vital for the proper interpretation of the obtained results.

   Overall, I consider this paper of high quality and can recommend it for publication after the authors address all the concerns listed.
